# Targeting chordoma via an isocitrate dehydrogenase-1-dependent susceptibility to redox metabolism

**DOI:** 10.1007/s00401-026-03048-9

**Published:** 2026-07-23

**Authors:** Matthew Pun, Akash Deogharkar, Siva Kumar Natarajan, Nicholas Nuechterlein, Wentao Tian, Sunjong Ji, Eleanor Young, Fusheng Yang, Debra Hawes, Anthony C. Andren, John Henry Owen, Sagar Rau, Fengyun Su, Xuhong Cao, Abhijit Parolia, Mark Prince, Joshua Fry, Paul A. Gardner, Rendong Yang, Alexander R. Judkins, Carl J. Koschmann, Andrew S. Venteicher, Costas A. Lyssiotis, Arul M. Chinnaiyan, Sriram Venneti

**Affiliations:** 1 Department of Pathology, Laboratory of Brain Tumor Metabolism and Epigenetics, University of Michigan, Ann Arbor, MI USA; 2 Department of Pediatrics, Michigan Medicine, Ann Arbor, MI USA; 3 Michigan Center for Translational Pathology, University of Michigan, Ann Arbor, MI USA; 4 Department of Pathology, Children’s Hospital of Los Angeles, Los Angeles, CA 90027 USA; 5 Department of Molecular and Integrative Physiology, University of Michigan, Ann Arbor, MI USA; 6 Department of Otolaryngology/Head Neck Surgery, University of Michigan School of Medicine, Ann Arbor, MI 48109-5616 USA; 7 Department of Pathology, University of Michigan, Ann Arbor, MI USA; 8 Howard Hughes Medical Institute, University of Michigan, Ann Arbor, MI USA; 9 Department of Urology, University of Michigan, Ann Arbor, MI USA; 10 Rogel Cancer Center, University of Michigan, Ann Arbor, MI USA; 11 Department of Urology, Northwestern University Feinberg School of Medicine, Chicago, IL 60611 USA; 12 Department of Neurological Surgery, University of Pittsburgh Medical Center, Pittsburgh, PA USA; 13 Robert H. Lurie Comprehensive Cancer Center, Northwestern University Feinberg School of Medicine, Chicago, IL 60611 USA; 14 Department of Neurosurgery and Center for Skull Base and Pituitary Surgery, University of Minnesota, Minneapolis, MN USA; 15 Department of Internal Medicine, Division of Gastroenterology, University of Michigan, Ann Arbor, MI USA

**Keywords:** Chordoma, IDH1, NRF2, Redox, Metabolism

## Abstract

Chordomas are rare cancers that arise along the axial skeleton. Alterations in metabolism are a hallmark of cancer, and we sought to identify metabolic vulnerabilities in chordoma. We discovered that the tricarboxylic acid (TCA)-related enzyme isocitrate dehydrogenase-1 (*IDH1*) was expressed highly in bulk and single-cell patient-derived chordomas and was associated with worse survival outcomes. IDH1 catalyzes the conversion of isocitrate and nicotinamide adenine dinucleotide phosphate (NADP+) to alpha-ketoglutarate (⍺-KG) and NADPH. This critical reaction influences TCA cycle metabolism, regulates epigenetic pathways, and affects redox balance. Both *IDH1* knockdown and treatment with an inhibitor targeting IDH1 were toxic to chordoma cells. An integrated analysis of the transcriptomic, chromatin, and metabolomic responses on IDH1 inhibition converged on deregulated glutathione metabolism. IDH1 inhibition was associated with increased expression and enrichment of activating H3K27ac at NRF2 (nuclear factor erythroid 2-related factor 2) signature genes including those in the glutathione biosynthetic pathway. This was accompanied by reduction of both NADPH/NADP+ and reduced/oxidized glutathione (GSH/GSSG) ratios. Importantly, IDH1 inhibitor-driven toxicity was rescued via media supplementation with the antioxidant *N*-acetylcysteine, suggesting that IDH1 inhibition in chordomas creates a redox-dependent metabolic vulnerability. Finally, IDH1 inhibitor treatment reduced tumor growth in two independent chordoma mouse xenograft models. Our findings suggest a potential therapeutic avenue for further exploration in chordoma.

**Supplementary Information:**

The online version contains supplementary material available at 10.1007/s00401-026-03048-9.

## Introduction

Chordomas are aggressive malignancies that arise along the spinal axis and are hypothesized to develop from remnants of the embryologic notochord [12, 25, 46]. These tumors are relatively radioresistant and present surgical challenges due to their anatomic location. There are no approved systemic therapies for this class of tumors [46], highlighting the need for additional research and innovative strategies to improve patient outcomes.

While alterations in metabolism are a hallmark of cancer, little is known about the regulation of metabolic pathways in chordoma and how these pathways are involved in tumor development or maintenance. In patients, chordoma tumors exhibit moderate uptake of the glucose analog fludeoxyglucose (FDG) in Positron Emission Tomography (PET) imaging relative to other solid tumors [31]. Prior laboratory work using cell-line models identified collagen supplementation as beneficial for chordoma cell line proliferation whereas hypoxia and lower glucose conditions showed minimal effects [32, 51]. Additional studies focusing on recurrence patterns have proposed the carbohydrate metabolism pathway and the enzyme asparagine synthetase as potential predictors of more aggressive tumor behavior [8, 40].

Most recently, two studies have shed additional light on dysregulation of metabolic pathways in chordoma. Data from mtDNA sequencing on a cohort of pediatric and adult chordoma tumors revealed a significant enrichment in nicotinamide adenine dinucleotide (NADH) complex gene mutations [28], a finding consistent with earlier small sample size data (n = 3) showing recurrent mutations in *MT-ND4* [18]. Additionally, dysregulation of cholesterol metabolism was identified as a key feature associated with chordoma tumor budding and progression in four independent cohorts [57]. Together, these findings provide a growing understanding of metabolism in chordoma, but beyond these experiments, data on how metabolism is rewired in chordoma are limited. This current study leverages comparisons of chordoma to its putative tissue of origin to identify potential metabolic vulnerabilities. Our results further underscore the potential importance of redox metabolism to chordoma biology.

## Methods

### Cell culture

CH22 cells were cultured in RPMI 1640 with 10% fetal bovine serum (FBS). UM-Chor1 and UM-Chor5 cells were cultured in a 4:1 preparation of IMDM:RPMI with 1% antibiotic–antimycotic, 0.2% prophylactic Plasmocin^®^, and 10% FBS. U-CH1 and U-CH2 were cultured in a 4:1 preparation of IMDM:RPMI with 10% FBS and an additional 1% of L-glutamine. All cells were adapted to this preparation of IMDM:RPMI for metabolite snapshot experiments. JHC7 cell were cultured in DMEM:F12 with 10% FBS for initial dose curves with IDH1 inhibitor treatment. For all other experiments, JHC7 cell were adapted to a 4:1 preparation of IMDM:RPMI with 10% FBS and an additional 1% of L-glutamine. Cells were grown in humidified incubators at 37 °C in 21% O_2_ and 5% CO_2_. Tissue culture plates and flasks were pre-treated with Collagen I (Gibco™, rat tail) for U-CH1 and U-CH2 cells, consistent with prior practice. All other cells were maintained without collagen coating.

### Animal experiments

Animal experiments were performed after approval from the University of Michigan Committee on Use and Care of Animals (#PRO00010599) and were conducted as per National Institutes of Health (NIH) guidelines for animal welfare. All animals utilized in these studies were housed in pathogen-free conditions as per IACUC guidelines with continuous access to both food and water in addition to veterinary care under standard temperature conditions. NOD-SCID-IL2R gamma chain-deficient (NSG) mice (NOD.Cg-*Prkdc*scid*Il2rg*tm1Wjl/SzJ, #005557, 4–6 weeks old) were used for all experiments.

Flank models of CH22 and U-CH1 were established by injecting 1 × 10^6^ cells in PBS mixed with equal volume of Matrigel in flanks of mice. Treatment was started after palpable tumors (∼200mm^3^) appeared, and tumor volumes were measured twice a week. IDH1 inhibitor was first diluted to 10 mM in DMSO and then diluted in PBS and injected intraperitoneally at a concentration of 10 mg/kg animal body weight.

### Cell lysate preparation and histone extraction

For whole-cell protein extraction, cells were washed with PBS, centrifuged and the pellet was re-suspended in RIPA lysis buffer (Thermo Fisher Scientific #8990) supplemented with a cocktail of protease (Sigma #P8340) and phosphatase (APExBIO #K1012) inhibitors under rotation at 4 °C for one hour. The lysate mix was centrifuged and whole-cell protein-containing supernatant was transferred to another tube.

Histone proteins were collected by acid extraction. Briefly, suspension cells were washed with PBS (Gibco #10,010–023), and, after centrifugation, the cell pellet was re-suspended with hypotonic lysis buffer (10 mmol/L Tris–HCl pH8.0, 1 mmol/L KCl, and 1.5 mmol/L MgCl_2_) supplemented with a cocktail of protease (Sigma #P8340) and phosphatase (APExBIO #K1012) inhibitors at 4 °C for 30 min under rotation. The cell nuclei were isolated by centrifugation at 10,000 g, the supernatant was discarded, and the pelleted nuclei were re-suspended with sulfuric acid (0.4 N H_2_SO_4_) and incubated under rotation at 4 °C overnight. After centrifugation at 16,000 g, the histone-containing supernatant was transferred to another tube, mixed with trichloroacetic acid (Sigma #T0699), and incubated on ice for 30 min. Next, the tube was centrifuged, supernatant discarded, and isolated histones in the tube were washed twice with ice-cold acetone with a 5-min centrifugation between each wash. Histones were air-dried at a room temperature and re-suspended with double distilled water. Histones and whole-cell protein lysates were quantified with Pierce BCA Protein Assay Kit (Thermo Fisher Scientific #23,225) reagent.

### Western blotting

Frozen pulverized tissues or cultured cells were lysed with 1X RIPA buffer containing protease and phosphatase inhibitor cocktail (Sigma-Aldrich). Lysates were centrifuged at 14,000 g for 20 min at 4 °C, and supernatants were harvested. Protein concentrations in supernatants or extracted histones were detected using the BCA Protein Assay (23,225, Pierce). Lysates were separated by SDS-PAGE on Novex 4–12% Bis–Tris gels (NP0321, Invitrogen), transferred to PVDF membranes, and blocked in 5% nonfat milk or 5% BSA in TBS containing 0.2% Tween 20. Membranes were probed with the following primary antibodies: anti-H3 Total (3638, Cell Signaling), anti-H3K27ac (07–360, Millipore Sigma), anti-H3K27me3 (9733, Cell Signaling), anti-IDH1 (8137, Cell Signaling Technology), anti-SLC7A11 (98,051, Cell Signaling Technology), anti-GCLM (33,381, Cell Signaling Technology), anti-GSR (62,448, Cell Signaling Technology), anti-vinculin (V9264, Sigma-Aldrich). Immunoreactivity was detected after probing the blots with horseradish-peroxidase-conjugated anti-rabbit/mouse secondary antibody (6515/6516, Bio-Rad) followed by SuperSignal West Pico PLUS Chemiluminescent Substrate using autoradiography film (dotScientific #BDB57-Lite) or iBright^™^ CL1500 Imaging System, (Invitrogen, Thermo Fisher Scientific cat#44,240).

### IDH1 knockdown via short hairpin RNA (shRNA)

Bacterial stocks of five MISSION® shRNA constructs targeting the coding sequence of IDH1 were obtained from Sigma-Aldrich with the following identification numbers: TRCN0000027249, TRCN0000027253, TRCN0000027284, TRCN0000027289, and TRCN0000027298. Bacteria were streaked onto ampicillin-containing agar plates and grown overnight at 37 °C. Single colonies were picked and cultured in Luria broth (LB) containing ampicillin with 250 rotations per minute (rpm) agitation at 37 °C. After 18 h, bacterial cultures were collected, and plasmid DNA was isolated using PureLinkTM HiPure Plasmid Maxiprep Kit (Thermo Fisher Scientific, K210007). DNA was quantified and assessed for purity with Agilent BioTek microplate reader.

Plasmids passing quality standards were used for lentivirus production. First, HEK293T cells were plated in DMEM media with 10% fetal bovine serum (FBS) but without antibiotics and grown 24 h to 70% confluence. At that time, preparation of 0.26 mL of serum-free DMEM, 6.7 μL of pPackH1 packaging plasmid, 0.67 μg of the shRNA-containing plasmid were combined for each transduction. To each solution, 8 μL of PureFection transduction reagent was added, and the mixtures were vortexed for 10 s and then incubated for 30 min. After incubation, plasmid mixtures were added to HEK293T cells dropwise with frequent mixing of media. Cells were then incubated overnight at 37 °C and 5% CO_2_. After 18–24 h, transduction media were replaced with DMEM with 1% penicillin–streptomycin. Resulting viral supernatants were collected at 48 and 72 h after transduction, pooled, filtered for cells, and then added to chordoma plated cell lines. Chordoma cells were incubated with virus-containing DMEM for 72 h, then returned to their respective media. Puromycin at 1 μg/mL was added to the chordoma cultures. This concentration was increased with subsequent passages if knockdown levels were not maintained, up to 4 μg/mL.

### IDH1 knockdown via small interfering RNA (siRNA)

MISSION^®^ siRNA Transfection Reagent (Sigma Aldrich S1452) and MISSION^®^ siRNAs (Sigma Aldrich) were used to knockdown IDH1. The manufacturer-provided protocol for forward siRNA transfection into adherent cells in 6-well plates was used for this experiment; each of the siRNA reactions was run in triplicates. Briefly, 150,000 cells per well in a 6-well plate were seeded in 3 mL of the antibiotic free culture media. The cells were incubated overnight at 37 °C. On the next day, mixes containing 175 μL of serum-free media per well, 15 μL transfection reagent per well, and 10 μL siRNA solution per well (final concentration 25 nM/well) were prepared for each siRNA and incubated for 15 min at room temperature. During incubation, old media were replaced with 2 mL of fresh antibiotic-free media per well. Then, 200 μL of each master mix was added dropwise to each well. 24 h after incubation at 37 °C, media containing the siRNA mix were replaced with 3 mL of fresh media and 72 h post-transfection and incubation at 37 °C, cells were counted and extracted for protein for subsequent western blotting to confirm siRNA-mediated knockdown of IDH1.

### Cell proliferation assays

Unless otherwise specified, cells were trypsinized and counted to seed 25,000 cells/well in 24-well plates. Cells were counted four days later. Each well underwent trypsinization, cell collection, and resuspension with trypan blue exclusion. Count estimates were made using Countess automated cell counters.

For nutrient withdrawal experiments, cells for seeding were re-suspended in PBS following trypsinization and collection to wash out residual complete media. Cells were then centrifuged and re-suspended in base media lacking glucose, sodium pyruvate, and glutamine. Dilutions for seeding were made into media with the final concentrations of glucose and/or glutamine per experimental design. Cells were then seeded in 1 mL of media per well in 24-well plates.

For experiments in lentivirally transduced cell lines, cells were plated in 1 mL of media per well containing puromycin. Cell counts of initial passages were tested in 1 μg/mL puromycin.

For pharmacologic treatment experiments, cells were plated at 25,000 cells/well in 500 μL of media per well. On the subsequent day, inhibitors were prepared in media at 2X concentrations and 500 μL of the resulting preparations were applied to each of the appropriate wells. Cells were counted 4 days after treatment.

For oxalomalate treatment, 3000 cells/ well were seeded in a 96-well plate, the luminescence was measured 72 h after treatment with different concentrations of oxalomalate (Cayman, #13,521), following the CellTiter-Glo kit protocol (Promega #G9241).

### Glycolysis and mitochondrial stress tests

Cells were treated with vehicle or IDH1 inhibitor seeding into XF96 cell culture microplates (Agilent Technologies #101,085–004) coated with CellTak (Corning #354,240) in XF DMEM (Agilent Technologies #103,575–100) for 6 to 8 replicates. For glycolysis stress tests, XF DMEM was supplemented with 2.5 mmol/L glutamine and 0.5 mmol/L sodium pyruvate (Agilent Technologies #103,579–100 and #103,578–100). For mitochondrial stress tests, XF base media were supplemented with 17.5 mmol/L glucose, 2.5 mmol/L glutamine, and 0.5 mmol/L sodium pyruvate (Agilent Technologies #103,577–100, 103,579–100, and 103,578–100). XFe96 sensor cartridges (Agilent Technologies #102,416–100) were loaded with glucose, oligomycin, and 2-deoxy-D-glucose for glycolysis stress tests (Agilent Technologies #103,020–100) or oligomycin, FCCP, and rotenone/antimycin A for mitochondrial stress tests (Agilent Technologies #103,015–100). Glycolysis and mitochondrial stress tests were performed with a Seahorse XFe96 Analyzer (Agilent Technologies) using standard drug injection and OCR and ECAR measurement protocols defined in the Seahorse Wave Desktop Software (Agilent Technologies). Results were normalized to the number of cells seeded immediately prior to assays.

### Bulk RNA expression analyses from published patient tumors

Microarray-based transcriptional analysis: Affymetrix GeneChip^®^ HG-U133 Plus 2.0 gene expression data generated by the Ramnik Xavier Lab were obtained from http://xavierlab2.mgh.harvard.edu/chordoma/Data.html. Expression values for notochord and chordoma samples were compared by a two-sided Student’s t test. Multiple testing correction was applied by the Benjamini–Hochberg method. For heatmap visualization, data were normalized across probes, and heatmaps were generated using Morpheus (https://software.broadinstitute.org/morpheus). Pathway analysis of differentially expressed genes was performed using Enrichr [7, 21, 49]. Gene-set enrichment analysis (GSEA) was performed first by generating ranked gene lists using the product of the negative log-10 transformation of the q-value and the difference in expression for each gene. A GSEA preranked analysis (GSEA 4.2.3) was then performed on the resulting gene lists with the following parameters: 15–500 as min–max gene set size; weighted scoring scheme; meandiv normalization; and Abs_max_of_probes mode.

Pediatric cBioPortal transcriptional analysis: RNA expression levels of *IDH1, IDH2, IDH3A,* and *IDH3B* were downloaded from tumor samples from the pediatric cBioPortal https://pedcbioportal.kidsfirstdrc.org/ [Chordoma Foundation Disease Models (Chordoma Foundation, Provisional) data set].

### Transcriptomic profiling of chordoma cell lines with IDH1 inhibition

Cells were plated in 10-cm dishes at a concentration designed to reach 60–80% confluence in 96 h. Initial seedings ranged between 5 × 10^5^ and 1 × 10^6^ cells/plate. After adhering within 24 h, cells were treated with 3 μM IDH1 inhibitor or a 1:10,000 dilution of dimethyl sulfoxide (DMSO) in the corresponding media. At 72 h post-treatment, cells were trypsinized, counted, and placed on ice. At least 500,000 cells/replicate were then lysed with 750 μL of QIAzol (Qiagen 79,306), vortexed for > 1 min, incubated at room temperature for 20 min and stored at −80 °C. Upon thawing of samples, RNA was extracted with chloroform and purified using the RNeasy (Qiagen) kit. Samples were evaluated for RNA integrity numbers (RIN). Libraries were generated from 200 to 1,000 ng of total RNA using KAPA RNA HyperPrep Kit with RiboErase (HMR). After RNA was fragmented to 200–300 bp with heat, double-stranded cDNA was synthesized, and New England Biolabs (NEB) adapters were ligated. Amplification was completed with KAPA HiFi HotStart mix and NEB dual barcodes were added according to manufacturer protocol. Agilent 2100 Bioanalyzer was utilized for quality control screening, and samples passing quality control were sequenced on an Illumina Novaseq X with 2 × 100 nucleotide read lengths and coverage of 15–20 million paired reads per sample. Psuedoalignment of reads to human (hg38/GRCh38) reference with Kallisto (v0.46.0) [4] was completed, and differential expression was determined with DEseq2 (v1.36.0)[23].

### Metabolite profiling of chordoma cell lines with IDH1 inhibition

For whole metabolite snapshot analysis, 1 × 10^6^ were plated in 10-cm dishes and cultured for 16 h at 37 °C in an incubator. Cells were then treated with DMSO or 3 μM of IDH1 inhibitor. Two days after treatment, a full medium change was performed with either DMSO or IDH1 inhibitor. Metabolites were then collected two hours later by the following process. Methanol (80%) was made freshly using HPLC-grade methanol (Sigma 34,860-1L-R) and water (Sigma 270,733-4L) and chilled to −80 °C. At a room temperature, all media were carefully aspirated from each plate such that no media remained. To each plate, 4 ml of dry ice-cold 80% methanol was added, and plates were immediately transferred to rest on dry ice. Once all plates received methanol, they were incubated at −80 °C. After 10 min, all cells were scraped off the dish and pipetted into 15-mL centrifuge conical tubes. Insoluble materials were pelleted at a maximum speed (> 3000 rpm) for 10 min at 4 °C, and supernatants were transferred to fresh 15-mL conical tubes. This step was repeated once, and samples were then stored at −80 °C. A fifth replicate for each condition was collected after trypsinization, lysed in RIPA buffer, and quantified using BCA protein quantification.

Methanol samples were thawed, and metabolites were pelleted by speedvac. Liquid chromatography coupled with tandem mass spectrometry (LC–MS/MS) metabolomics analysis was performed as described previously [22]. In brief, Agilent 1290 UHPLC and 6490 Triple Quadrupole (QqQ) Mass Spectrometer (LC–MS) were used for label-free targeted metabolomics analysis. Agilent MassHunter Optimizer and Workstation Software LC–MS Data Acquisition for 6400 Series Triple Quadrupole B.08.00 was used for standard optimization and data acquisition. Agilent MassHunter Workstation Software Quantitative Analysis Version B.0700 for QqQ was used for initial raw data extraction and analysis. For each MRM transition, its retention time of left delta and right delta of 1 min was used. Additional parameters include mass extraction window of 0.05 Da right and left from the extract m/z, Agile2 integrator algorithm, peak filter of 100 counts, noise algorithm RMS, noise SD multiplier of 5 min, S/N 3, Accuracy Max 20% max %Dev, and Quadratic/Cubic Savitzky-Golay smoothing algorithm with smoothing function width of 14 and Gaussian width of 5.

For reversed-phase liquid chromatography (RPLC), a Waters Acquity UPLC BEH TSS C18 column (2.1 × 100 mm, 1.7 μm) was used in the positive ionization mode with mobile phase (A) consisting of 0.5 mM NH4F and 0.1% formic acid in water; mobile phase (B) consisting of 0.1% formic acid in acetonitrile. Gradient program: mobile phase (B) was held at 1% for 1.5 min, increased to 80% in 15 min, then to 99% in 17 min and held for 2 min before going to initial condition and held for 10 min. For hydrophilic interaction liquid chromatography (HILIC), a Waters Acquity UPLC BEH amide column (2.1 × 100 mm, 1.7 μm) was used in the negative ionization mode with mobile phase (A) consisting of 20 mM ammonium acetate (NH4OAc) in water at pH 9.6; mobile phase (B) consisting of acetonitrile (ACN). Gradient program: mobile phase (B) was held at 85% for 1 min, decreased to 65% in 12 min, then to 40% in 15 min and held for 5 min before going to the initial condition and held for 10 min.

Both columns were at 40 °C and 3 μl of each sample was injected into the LC–MS with a flow rate of 0.2 ml/min. Calibration was achieved through Agilent ESI-Low Concentration Tuning Mix. Optimization was performed on the 6490 QqQ in the RPLC-positive or HILIC-negative mode for each of 245 standard compounds (215 and 217 compounds for RPLC-positive and HILIC-negative, respectively) to obtain the best fragment ion and MS parameters such as fragmentation energy for each standard. Retention time (RT) for each standard was measured from a pure standard solution or a mix standard solution. The LC–MS/MS methods were created with dynamic MRM (dMRM) with RTs, RT windows, and transitions of all 245 standard compounds. Key parameters of electrospray ionization (ESI) in both the positive and the negative acquisition modes are: Gas temp 275 °C, Gas Flow 14 l/min, Nebulizer at 20 psi, SheathGasHeater 250 °C, SheathGasFlow 11 l/min, and Capillary 3000 V. For MS: Delta EMV 200 V or 350 V for the positive or negative acquisition mode, respectively, and Cycle Time 500 ms and Cell Acc 4 V for both modes.

Pre-processed data with Agilent MassHunter Workstation Software Quantitative Analysis were post-processed for further quality control in the programming language R. First, we examined the distribution of sums of all metabolite abundance peak areas across individual samples in a given experiment as a measure for equal sample loading into the instrument. Next, we calculated coefficients of variation (CVs) in all biological replicate groups for each metabolite given a cut-off value of peak areas in each of the RPLC and HILIC methods. We then compared distributions of CVs for the whole dataset for a set of peak area cut-off values of 0, 1000, 5000, 10,000, 15,000, 20,000, 25,000, and 30,000 in each method. The noise cut-off value of peak areas in each method was chosen by manual inspection of the CV distributions. The noise-filtered data of individual samples were then normalized by the total intensity of all metabolites. We retained only those metabolites with at least two technical replicate measurements for a given experimental variable. Then, each metabolite abundance level in each sample was divided by the mean of all abundance levels across all samples in a given experiment for comparisons, statistical analysis, and visualizations among metabolites. This normalization and scaling method has been used in previous studies with biologically meaningful results [13]. Pathway analysis was done using the webtool, MetaboAnalyst (https://www.metaboanalyst.ca/).

### Chromatin immunoprecipitation followed by high-throughput sequencing of chordoma cell lines with IDH1 inhibition

Chordoma cell lines treated with either DMSO vehicle or 3 μM IDH1 inhibitor for three days were fixed according to the Active Motif Epigenetics Services ChIP Cell Fixation Protocol. First cells were fixed with 1% formaldehyde (Sigma #F-8775) in media, rocking for 15 min and quenched with 0.125 M glycine. Cells were next washed with a PBS-Igepal solution and then finally washed with 1 mM Phenylmethanesulfonyl fluoride (Sigma #P-7626) in PBS-Igepal before snap-freezing the pellets on dry ice. As performed by Active Motif, chromatin was then isolated by the addition of lysis buffer and disruption with a Dounce homogenizer. Lysates were sonicated and the DNA sheared to an average length of 300–500 bp. Chromatin aliquots were processed with RNase, proteinase K and heat for de-crosslinking. The resulting ethanol-treated precipitates were re-suspended and quantified via NanoDrop spectrophotometer to determine chromatin yields. Subsequently, 30 μg of chromatin per replicate was precleared with protein A agarose beads (Invitrogen). Genomic DNA regions of interest were isolated using 4 µg of antibody against H3K27ac (Active Motif). Next immunoprecipitants were washed, eluted from the beads in an SDS buffer, and subjected to RNase and proteinase K treatment. Crosslinks were reversed by incubation overnight at 65 °C, and ChIP DNA was purified by phenol–chloroform extraction and ethanol precipitation. Quantitative PCR (QPCR) reactions were carried out in triplicate on specific genomic regions using SYBR Green Supermix (Bio-Rad) for quality assessment. An automated system (Apollo 342, Wafergen Biosystems/Takara) was employed to generate Illumina sequencing libraries via end-polishing, dA-addition, adaptor ligation, and PCR amplification. Resulting libraries were quantified and sequenced on an Illumina NextSeq 500 (75 nt reads, single-end). Raw reads were aligned to the human reference genome (hg38) using the BWA algorithm (default settings). Reads were de-duplicated and screened for mapping quality >= 25. H3K27ac peak locations were determined using the MACS algorithm (v2.1.0) with a cutoff of p-value = 1e-7. Peaks that were on the ENCODE blacklist [2] of known false ChIP-seq peaks were removed. Spike-in of chromatin from another species, the fruit fly *Drosophila*, was performed and the number of test tags were normalized by the same number of spike-in *Drosophila* tags for each sample. Alignments were extended in silico at their 3’-ends to a length of 200 bp, which is the average genomic fragment length in the size-selected library and then assigned to 32-nt bins along the genome for visualization and stored in bigWig files. ChIP-seq signal at promoter and enhancer sites was assessed using deepTools (v3.5.1) computeMatrix and plotHeatmap tools [36]

### Reduced-to-oxidized glutathione quantification

Cells were plated at 2,500 (CH22) or 5,000 (UM-Chor1) cells/well into white-bottom 96-well plates and treated with IDH1 inhibitor at 3 μM for 24 or 48 h. Subsequently, cells were processed with the GSH/GSSG-Glo Assay (Promega, V6611) which quantifies total and oxidized glutathione. Ratios were calculated from luminescent intensity and compared between IDH1 inhibitor-treated and vehicle-treated cells.

### NADP/NADPH ratio measurement

For NADP^+^/NADPH ratios, 250,000 cells per well in a 6-well plate were seeded in 3 ml of the culture media and incubated overnight at 37 °C. Next day the cells were treated with 3 μM IDH1 inhibitor and after 48 h of incubation at 37 °C, processed for NADP^+^ and NADPH estimation following the protocol as described by the manufacturer (Promega, G9082). Briefly, following IDH1 inhibitor treatment, the 6-well plate was removed from the incubator and immediately placed on ice. The media were aspirated, and each well was rinsed with ice-cold PBS. 500 μL of ice-cold 1:1 mixture of PBS and 1% DTAB (dodecyltrimethylammonium bromide) in 0.2 M NaOH was added to each well and the cells were scraped off; the mixture was taken in an Eppendorf tube and split into two tubes of 200 μL each. In one tube, 100 μL of HCl was added and both the tubes were kept at 60 °C heating block for 15 min followed by 10 min at a room temperature. 100 μL of 0.5 M Tris was added to the tube containing 100 μL of HCl (NADP^+^ estimation) and 200 μL of 1:1 mixture of HCl and 0.5 M Tris was added to the second tube (NADPH estimation). 50 μL of the mixture was transferred to each well of the white-bottom 96-well plate in triplicates and 50 μL of the luminescence solution from the kit was added to each well. NADP+ and NADPH standards were prepared in PBS, 1% DTAB in NaOH, HCl and 0.5 M Tris solution. The 96-well plate was incubated at RT for 45 min and luminescence was measured using the Cytation 5 reader and NADP+/NADPH ratio was calculated.

### N-Acetyl-cysteine proliferation rescue experiments

Chordoma cells were plated as described above for the cell count experiments. In addition to the primary treatment of IDH1 inhibitor, either PBS or *N*-acetylcysteine at a final concentration of 5 mM was added to the media for treatment.

### Single-cell RNA-seq data and preprocessing

Pre-filtered single-cell RNA-seq (scRNA-seq) data from six newly diagnosed chordoma tumors published by Arrieta et al*.* [3] were downloaded from Zenodo (10.5281/zenodo.14885380; associated manuscript 10.1093/neuonc/noaf213), data obtained on October 24, 2025.

Each tumor was analyzed separately with Scanpy (version 1.11.5) in Python 3.11. For each tumor, further quality control metrics were computed with sc.pp.calculate_qc_metrics: cells with fewer than 200 detected genes and genes expressed in fewer than 3 cells were removed. No additional filtering based on mitochondrial read fraction or doublet detection was applied beyond that performed by Arrieta et al*.* Raw counts were preserved for downstream visualization and pseudobulk aggregation.

2000 highly variable genes (HVGs) were identified from the raw counts using sc.pp.highly_variable_genes with Scanpy’s Seurat v3 flavor. Library-size normalization was then performed, followed by natural log transformation (log(x+1)). 

For dimensionality reduction, the expression matrix was restricted to HVGs. Gene expression values were scaled to unit variance and centered, with values clipped at an absolute value of 10. Principal component analysis (PCA) was performed, and a k-nearest-neighbor graph was computed. UMAP embeddings were calculated with sc.tl.umap, and Leiden clustering was performed using sc.tl.leiden with flavor = 'igraph', n_iterations = 2, and directed = False. Clustering and UMAP were performed separately for each tumor. GRCh38 was used throughout.

### Copy-number inference and neoplastic vs. non-neoplastic annotation of scRNA-seq data

For each tumor, large-scale somatic copy-number alterations (SCNAs) were inferred using the Python package infercnvpy (version 0.6.1). Genes were ordered by genomic coordinates from GRCh38, and copy-number inference was performed with a sliding window of 250 genes and default infercnvpy parameters unless otherwise noted.

Clusters were annotated as neoplastic (tumor), non-neoplastic (normal), or mixed by integrating TBXT expression with SCNA burden. TBXT expression, which is typically restricted to chordoma tumor cells and absent from adjacent normal tissues [45], was used as a primary marker of neoplastic identity. Clusters with high TBXT expression and broad SCNA shifts were annotated as neoplastic. Clusters with low TBXT expression and no detectable SCNA shifts were annotated as non-neoplastic. Clusters with intermediate SCNA burden and/or heterogeneous TBXT expression were annotated as “mixed”. All downstream tumor-specific analyses used cells from clusters annotated as neoplastic; mixed clusters were excluded.

### Metabolic gene signature scoring in scRNA-seq

To assess metabolic heterogeneity within chordoma tumors, we defined a TCA-cycle-related metabolic gene signature consisting of 27 genes: *DLAT, DLD, DLST, FH, IDH1, IDH2, IDH3A, IDH3B, IDH3G, MDH1, MDH2, ACLY, ACO1, OGDH, ACO2, PC, PCK1, PCK2, PDHA1, PDHA2, PDHB, SDHB, SDHC, SDHD, SUCLG2, SUCLG1,* and* SUCLA2.*

For each tumor, log-normalized expression values were z-scored per gene across all neoplastic cells. The metabolic gene signature score for each cell was defined as the mean z-score across the 27 genes. This continuous signature was then visualized on UMAP coordinates for each tumor.

### IDH1 and IDH2 expression calling in single cells

For the analysis of IDH1 and IDH2 expression frequencies, cells were classified as “IDH1-expressing” or “IDH2-expressing” if the log-normalized expression value for the respective gene was greater than 0 (i.e., at least one non-zero count prior to log transformation); cells with values of 0 were classified as non-expressing. This binary “expressed vs. not expressed” status was used both for UMAP colorings and for calculating the proportion of IDH1-expressing and IDH2-expressing tumor cells in each tumor.

### Pseudobulk differential expression from scRNA-seq and KEGG pathway analysis

To identify transcriptional programs associated with IDH1 expression in tumor cells, we generated pseudobulk profiles from the single-cell data. For each tumor, raw counts from neoplastic cells were summed separately for IDH1-expressing and non-expressing cells, yielding 12 pseudobulk samples (6 tumors × 2 IDH1 status groups). Only genes expressed in at least 10 pseudobulk samples were retained.

Differential expression between IDH1-expressing and non-expressing pseudobulk samples was performed using PyDESeq2 (version 0.5.3) in Python (version 3.11). A negative binomial generalized linear model was fitted with design formula “ ~ patient + IDH1_status”, comparing IDH1-expressing versus non-expressing pseudobulks. Genes with Benjamini–Hochberg adjusted p-value < 0.05 were considered significantly differentially expressed.

For pathway analysis, KEGG gene sets were interrogated using the R package clusterProfiler (version 4.14.0). When gene-level log2 fold change values were available, gene-set enrichment analysis (GSEA) was performed using gseKEGG, ranking genes by log2 fold change. Where only gene lists were used (e.g., for selected upregulated or downregulated genes defined by FDR and log2 fold change thresholds), over-representation analysis was performed using Enrich KEGG on upregulated and downregulated genes separately. Only KEGG pathways with false discovery rate (FDR) < 0.05 were retained for visualization.

### Statistical analysis

Unless otherwise specified, statistical analysis was performed in R version 4.4 and Python version 3.11. For differential expression of RNA-seq and pseudobulk data, DESeq2 or PyDESeq2 was used with Benjamini–Hochberg correction for multiple testing.

### Quantification and statistical analysis

Data are plotted as the means ± standard deviation (S.D.). Prism software (versions 10 Graphpad) was used to plot and analyze. Each figure legend contains the sample size (*n*) along with the statistical test performed. *P*-values are indicated in each figure or figure legend. Unpaired, two-tailed, two-sided, Student’s *t* test or analysis of variance (ANOVA) was used to analyze data. Z-scores were calculated by subtracting each number in a given analysis by the overall average and dividing by the standard deviation. Survival analysis was performed using Kaplan–Meier analysis with the log-rank test. Data were considered significant if *P-values* were below 0.05 (95% confidence intervals). Multivariate analysis was performed using Cox regression method.

## Results

### IDH1 expression is elevated in chordomas and associated with poor clinical outcomes

**Fig. 1:**
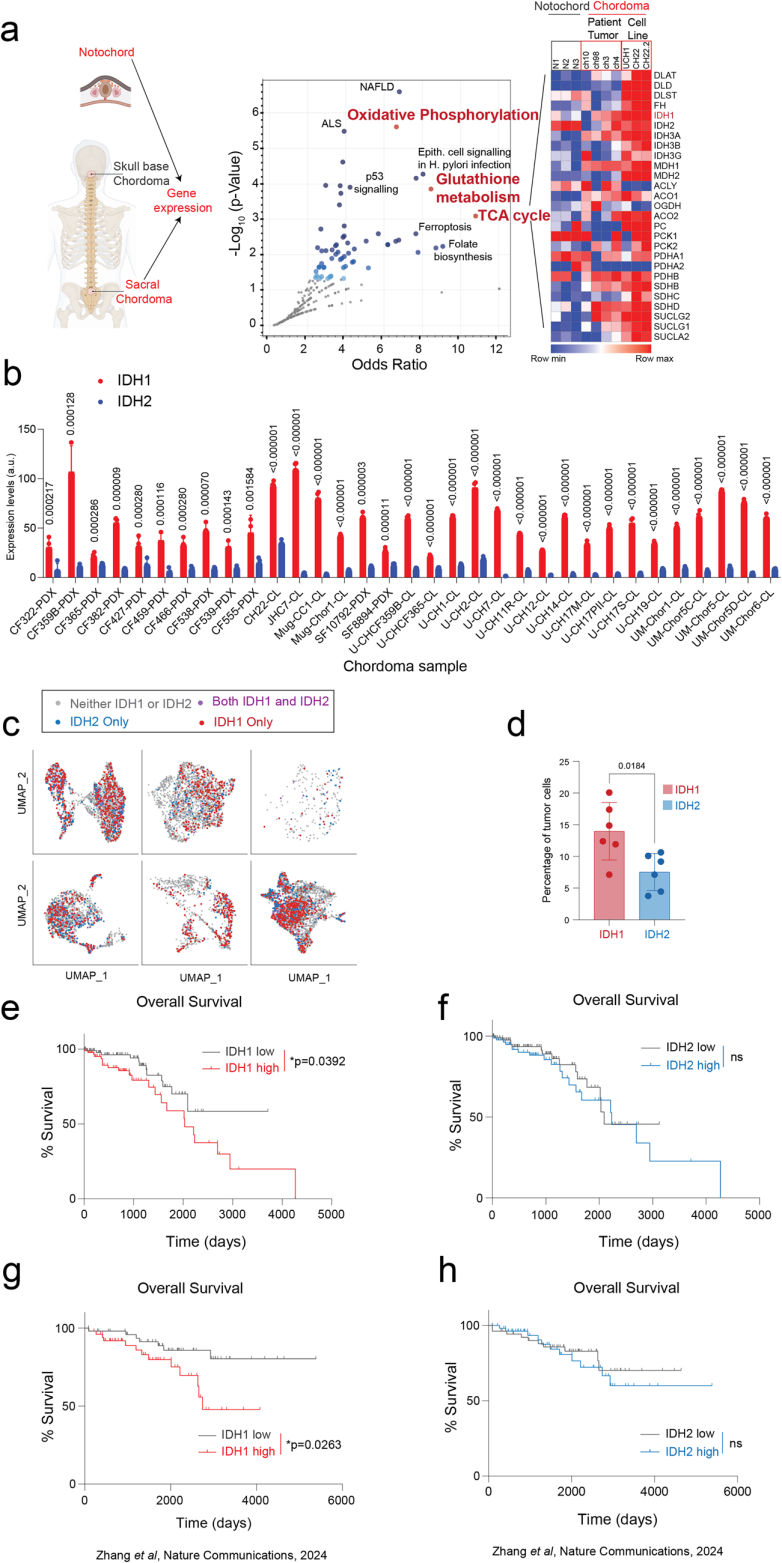
*IDH1* expression is elevated in chordomas and associated with poor clinical outcomes. **a** Schematic outlining the sources of gene expression data from the Ramnik Xavier lab (http://xavierlab2.mgh.harvard.edu/chordoma/Data.html) compared to known chordoma sites and graphs displaying microarray expression data enrichment results for metabolic pathways in chordoma tumors (*n* = 4) and cell lines (*n* = 3) when compared to notochord (putative tissue of origin, n = 3). Negative Log_10_ *P*-value (Y-axis) and Odds ratio (X-axis) are plotted. **b** Expression levels (Y-axis, a.u.) of the two predominant isocitrate dehydrogenases (IDH1 and IDH2) isoforms across chordoma samples (X-axis, n = 33) from data obtained from the PedcBioPortal. **c** UMAP projections of neoplastic cells labeled by IDH1 and IDH2 expression status (IDH1 only, IDH2 only, both, or neither). **d** Percentage of neoplastic cells expressing IDH1 or IDH2 in six tumors, demonstrating significantly higher frequency of IDH1 expression. Each point represents one tumor. **e** and **f** Kaplan–Meier plot comparing probability (Y-axis) of overall survival over time in days (X-axis) for chordoma patients with either high (red) or low (black) expression of *IDH1* (**e**) and *IDH2* (high—blue and low—black) (**f**) in a cohort of skull-base chordoma patients, stratified by median expression level (*n* = 181). **g** and **h** Kaplan–Meier plot comparing probability (Y-axis) of overall survival over time in days (X-axis) for chordoma patients with either high (red) or low (black) expression of *IDH1* (**g**) and *IDH2* (high—blue and low—black) (**h**) using data obtained from Zhang et al. (2024), stratified by median expression level (*n* = 104). Data are plotted as means ± SD in **d** and analyzed by unpaired Student’s *t* test. Data in **e**–**h**, analyzed by Log-rank test with 95% c.i

We performed an initial survey of gene expression patterns in sacral chordoma tumor samples and cell line models compared to control notochord tissue from publicly available gene expression microarray data. Gene-set enrichment analysis (GSEA) of upregulated genes in chordoma identified several metabolic pathways. These included oxidative phosphorylation (OxPhos), glutathione metabolism, and the tricarboxylic acid (TCA) cycle, among others (Fig. 1a). Single-cell RNA-seq data from six publicly available chordoma tumor samples demonstrated heterogeneous expression and showed a subset of tumor cells enriched for TCA cycle expression which also aligned with TBXT expression (Sup. Figs S1a–d). A more detailed evaluation of the genes within the TCA cycle KEGG pathway revealed increased expression of isocitrate dehydrogenase (IDH) family members (Fig. 1a). From PedcBioPortal data, gene expression studies of chordoma samples (n = 33) showed a higher expression of *IDH1* compared to other *IDH* isoforms (*IDH2, IDH3A, and IDH3B*, Fig. 1b and Sup. Fig. S1e, f). Similarly, the single-cell RNA-seq data from chordoma tumor samples showed greater numbers of tumor cells expressing IDH1 versus IDH2 (Fig. 1c–d).

Recurrent mutations of NADP^+^-dependent *IDH1 and IDH2* isoforms have been identified in glioma, acute myeloid leukemia, and chondrosarcoma [1, 24, 33]. Small-molecule inhibitors of both IDH1/2 mutations (IDH1/2 m) have been approved by the FDA for treatment of gliomas and myeloid leukemias. Moreover, recent studies have demonstrated that targeting wildtype IDH1 enzyme as a promising therapeutic strategy across several tumor types [9, 44, 53, 54]. Assessment of *IDH1* expression patterns in a cohort of skull-base chordoma samples (*n* = 181) demonstrated that high versus low *IDH1* expression (above or below median level of expression, respectively) was significantly associated with a poor outcome (Fig. 1e and Sup. Figs. S2a–b). Although this cohort was largely restricted to skull-base chordomas, there were no significant differences between the IDH1-high and -low cohorts in terms of patient age, specific tumor site, extent of resection, or metastatic status (Sup. Figs. Sc–f). In multivariate analysis with these covariates, IDH1-high expression was still statistically significantly associated with worse PFS but not with OS outcomes (Table S1).

In contrast, stratification based on median expression of *IDH2* revealed no significant difference with regards to overall survival (OS), though the *IDH2*-high cohort was significantly associated with worse progression-free survival (PFS) (Fig. 1f and Sup. Fig S2h). In an independent, previously published patient cohort, this association of high *IDH1* expression with worse overall and progression-free survival was confirmed, whereas *IDH2* gene expression was not associated with OS or PFS outcomes (Fig. 1g, h and Sup. Figs. S2h-i) [50]. In this cohort, chromosome 1q gain has been associated with poor survival outcomes, and 9p and 10q loss were previously associated with immune escape. There were no significant differences in frequency of each of these chromosomal alterations between the *IDH1*-high and -low subgroups (Sup. Figs. S2j-l). Next, we used Cox proportional hazards models to test the clinical significance of all the TCA cycle-related genes from Fig. 1A and again, IDH1 stood out as the most clinically significant gene for progression-free survival (Sup Fig. S2m). Therefore, we focused our efforts on targeting IDH1 in chordomas.

Because the TCA cycle is dependent on both glucose and glutamine as carbon sources, we tested the effect of withdrawal of these nutrients from cell-culture media in a panel of chordoma cell lines. All cells showed sensitivity to withdrawal of both glucose and glutamine to varying levels (Sup. Fig. S2n). Throughout our study, we utilized chordoma cell lines derived from both major tumor sites (sacral and clival), both sexes, a range of ages, all stages of disease, and varying degrees of previous treatment exposure (Table S2).

### Targeting IDH1 is toxic to chordoma cells

**Fig. 2:**
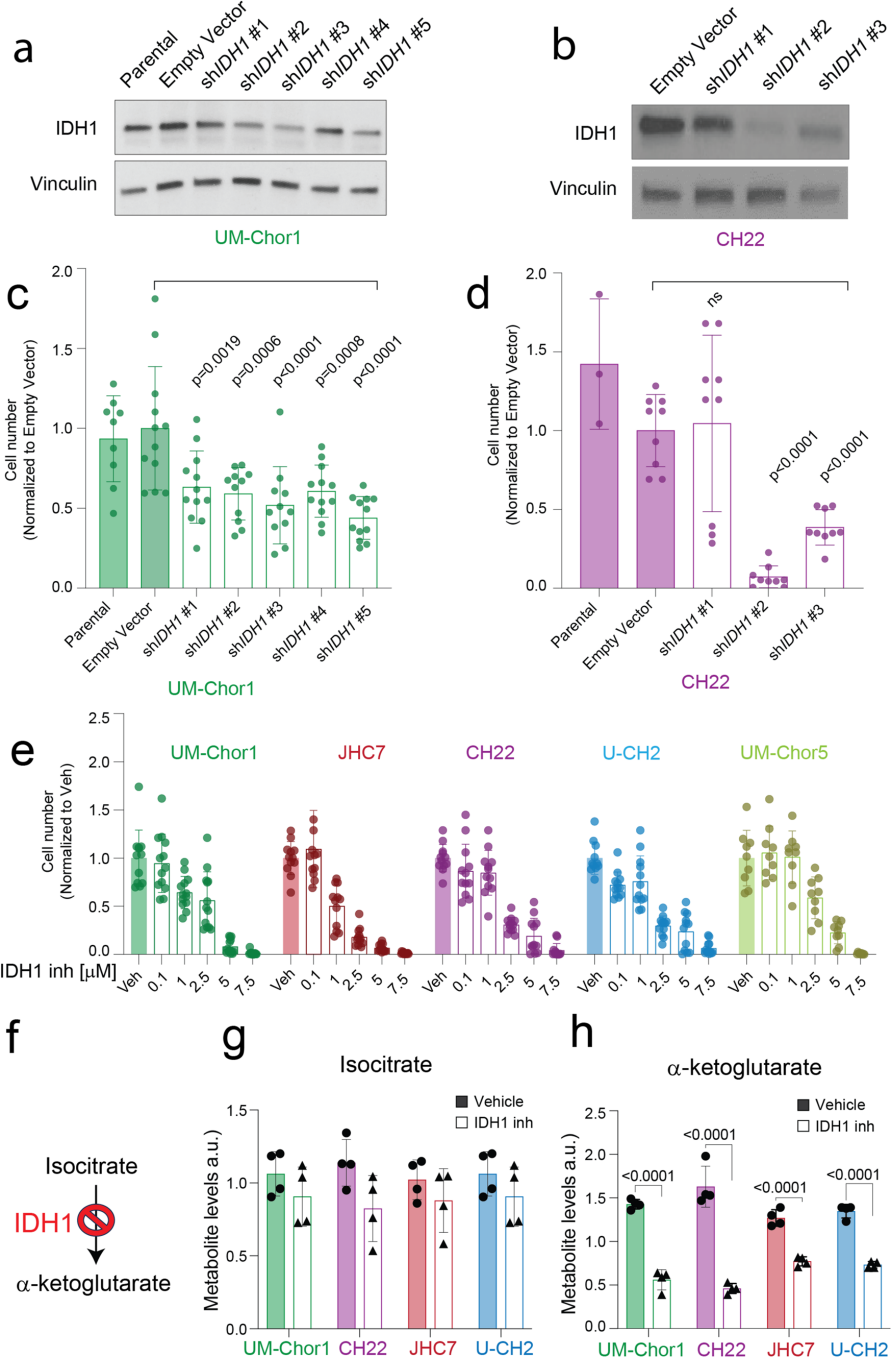
Targeting IDH1 is toxic to chordoma cells. **a** Representative protein immunoblots for IDH1 (top) and the loading control vinculin (bottom) in the UM-Chor1 chordomas transfected with empty vector or five independent IDH1 shRNA. **b** Representative protein immunoblots for IDH1 (top) and the loading control vinculin (bottom) in the CH22 chordomas transfected with empty vector or three independent IDH1 shRNA. **c** Cell counts (normalized to empty vector control, Y-axis) in UM-Chor1 cells with or without shIDH1 from Fig. 2a (*n* = 3 separate experiments with 3–4 replicates per trial). **d** Cell counts (normalized to empty vector control, Y-axis) in CH22 cells with or without shIDH1 from Fig. 2b (*n* = 3 separate experiments with 3–4 replicates per trial). **e** Cell counts normalized to a vehicle (DMSO control, Y-axis) across indicated increasing concentrations of IDH1 inhibitor evaluated in five chordoma cell-line models (X-axis) four days after treatment. **f** IDH1 canonically catalyzes a reaction converting isocitrate into α-KG. **g** Relative concentration of isocitrate (a.u., Y-axis) measured across four indicated chordoma cell lines (*n* = 4 replicates, each) treated with vehicle (DMSO) or 3 µM IDH1 inhibitor for 48 h prior to metabolite isolation. **h** Relative concentration of α-KG (a.u., Y-axis) measured across four indicated chordoma cell lines (*n* = 4 replicates, each) treated with vehicle (DMSO) or 3 µM IDH1 inhibitor for 48 h prior to metabolite isolation. Data analyzed by unpaired, two-sided, two-tailed Student’s t test with 95% c.i. Data are plotted as mean ± SD. Data analyzed in c–d using ANOVA, and in g–h by 2-sided, unpaired, 2-tailed, Student’s t test, all with 95% confidence intervals

We knocked down *IDH1* using multiple independent short-hairpin RNA (shRNA) and small interfering RNA (siRNA) in chordoma cell lines UM-Chor1 and CH22. Levels of shRNA knockdown varied by construct and by cell line transduced (Fig. 2a, b). Nevertheless, IDH1 knockdown was associated with decreased cell proliferation across both chordoma models (Fig. 2c, d). In an orthologous manner, siRNA-mediated reduction of IDH1 expression in UM-Chor1 and CH22 also resulted in reduced cell proliferation (Sup. Figs. 3a, d). We pharmacologically suppressed IDH1 with a wild-type inhibitor previously assessed in other tumor cell lines and xenograft models (IDH1i) [9]. Similar to genetic IDH1 knockdown, chordoma cell lines were sensitive to treatment with IDH1i, exhibiting IC_50_ values in the low µM range across all cell lines tested (Fig. 2e).

**Fig. 3:**
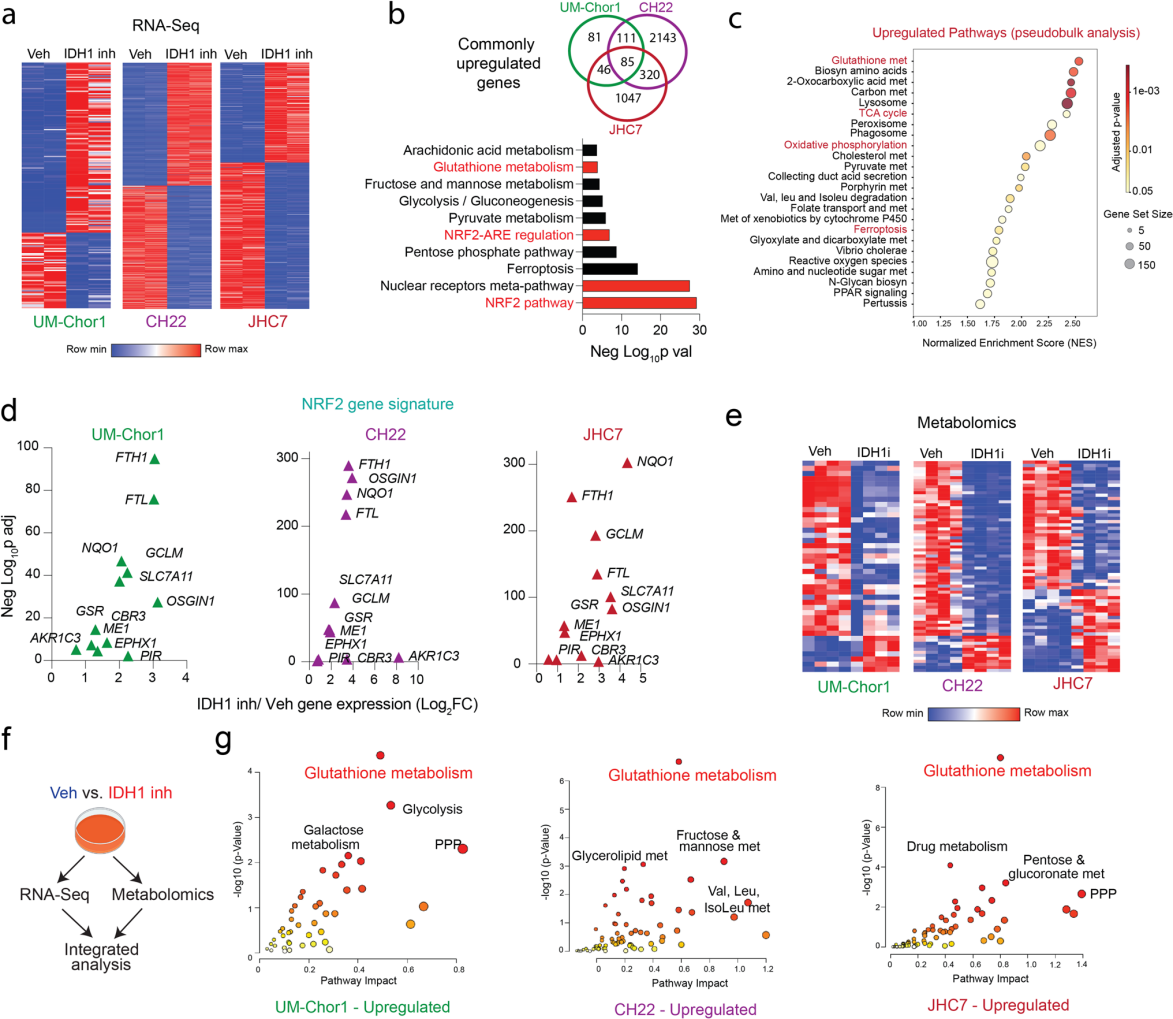
Glutathione metabolism is a key response pathway to IDH1 inhibition. **a** Heatmaps of differentially regulated genes between IDH1 inhibitor treatment and vehicle control for the chordoma cell lines, from left to right, UM-Chor1, CH22, and JHC7 (n = 2 replicates each). **b** Top: Three-way Venn diagram showing overlap of genes upregulated (DESeq2, FDR < 0.05) following IDH1 inhibition across three chordoma cell lines. The central intersection identifies genes consistently upregulated by IDH1 inhibition. Bottom: Gene set enrichment analysis (GSEA) of upregulated genes of the overlapping 85-gene gene set from the three tested chordoma cell lines with pathways illustrated along the Y-axis (negative Log_10_ *P*-value) for each pathway long the X-axis. **c** KEGG gene-set enrichment analysis of genes upregulated in IDH1-expressing tumor cells showing enrichment for oxidative phosphorylation, TCA-cycle, and glutathione metabolism pathways. **d** Scatter plots (Y-axis negative Log_10_ *P*-value versus fold change, and X-axis Log2FC) illustrating expression of NRF2 target signature gene expression in IDH1i vs. vehicle-treated UM-Chor1 (green), CH-22 (pink) and JHC7 (purple). **e** Heatmaps of differentially regulated metabolites between IDH1 inhibitor treatment and vehicle control for, from left to right, UM-Chor1, CH22, and JHC7 (4 replicates, each). **f** Schematic illustrating the integration of metabolomic and RNA-seq analysis of three chordoma cell lines treated with IDH inhibitor versus vehicle. **g** Integrated transcriptomic and metabolic pathway impact analysis of upregulated metabolic pathways for, from left to right, UM-Chor1, CH22, and JHC7 comparing pathway impact (X-axis) versus negative Log_10_ *P*-value (Y-axis)

IDH1 metabolizes isocitrate to α-KG (Fig. 2f) and treatment with this inhibitor resulted in significant depletion of α-KG, but not isocitrate levels across multiple chordoma models tested (Fig. 2g, h). No consistent changes in upstream or downstream metabolites citrate, succinate, and malate were observed with IDH1i treatment across all four cell lines (Sup. Figs. S3e–g). Moreover, Seahorse analysis did not reveal any marked changes in oxygen consumption rates (OCR) or extracellular acidification rates (ECAR) suggesting no major changes in TCA cycle metabolism or glycolysis on IDH1 inhibition (Sup. Figs. S3h–i).

### Glutathione metabolism is a key response pathway to IDH1 inhibition

To assess downstream functional pathways on IDH1 inhibition, we performed both RNA-sequencing (RNA-seq) and metabolic assays in three independent chordoma cell lines. RNA-seq in each cell line showed both upregulation and downregulation of several genes (Fig. 3a) (Table S3-5). We identified commonly upregulated and downregulated genes across all three cell lines on IDH1 inhibition (Fig. 3b, Sup. Fig. S4a) (Table S6-7). GSEA of upregulated genes shared across these cell lines showed enrichment of metabolic signatures including NRF2 (nuclear factor erythroid 2-related factor 2)-related pathways and glutathione metabolism (Fig. 3b) (Table S8) [28]. Enrichment analysis of downregulated genes was notable for axon guidance, adhesion, and tight junction-related pathways (Sup. Fig. S4b) (Table S9). Brachyury (*TBXT)* is a critical, upregulated transcription factor in chordomas [14, 35, 38, 41] and its epigenetic regulation has been proposed as a potential therapeutic vulnerability [10]. We ruled out significant changes in *TBXT* expression among all three cell lines after IDH1 inhibition (Sup. Fig. S4c). Differential expression analysis of pseudobulk profiles of the single-cell data stratified by *IDH1* status revealed widespread transcriptional changes between *IDH1*-expressing and non-expressing tumor cells (Sup. Fig. S4d). KEGG gene-set enrichment analysis of genes upregulated in *IDH1*-expressing tumor cells shows strong enrichment for, as expected, TCA cycle and oxidative phosphorylation. This analysis also identified glutathione metabolism among the most significantly upregulated pathways (Fig. 3c) (Table S10). KEGG analysis of the downregulated genes in *IDH1*-expressing cells revealed immune response-related pathways (Sup. Fig. S4e) (Table S11).

**Fig. 4:**
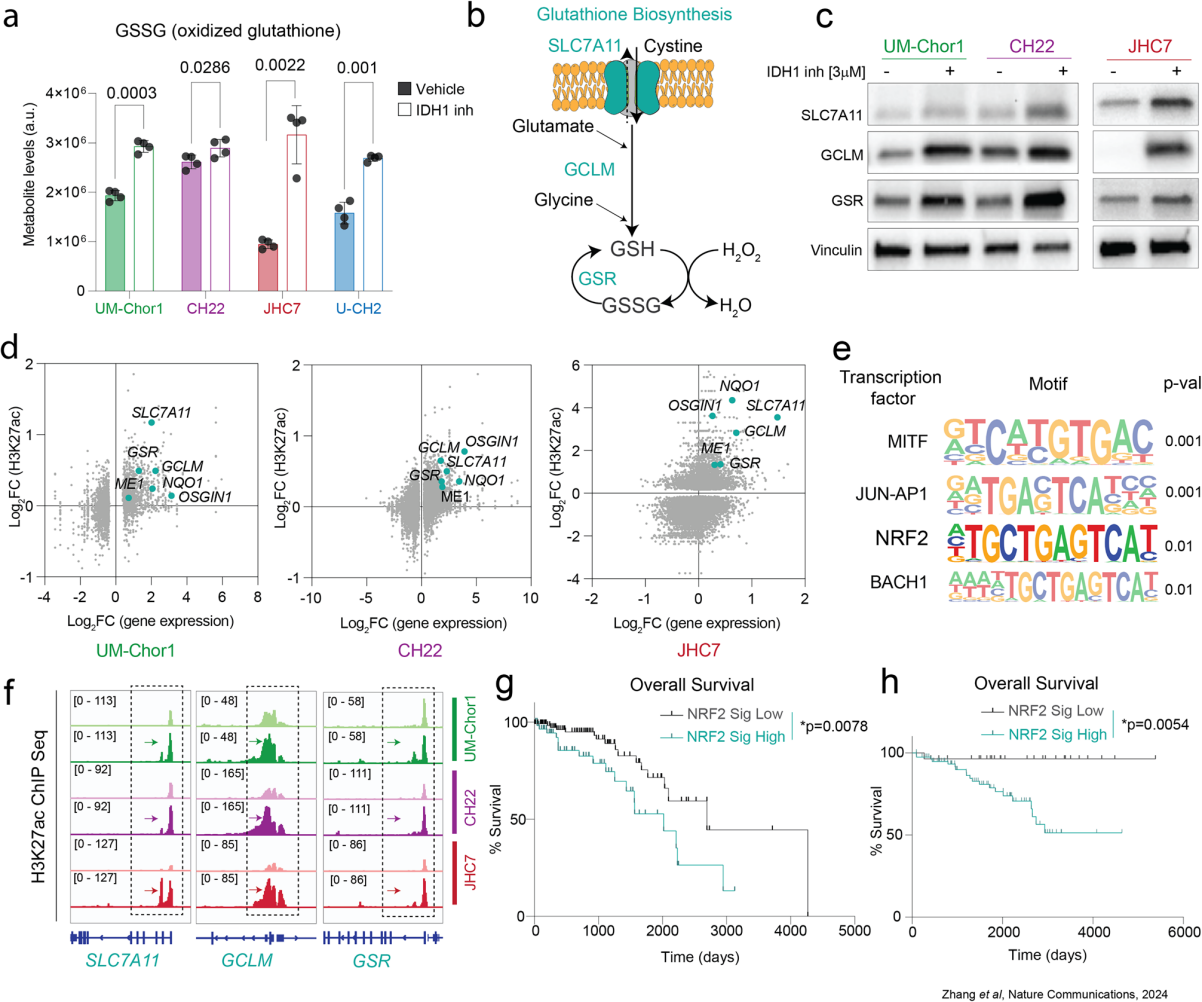
IDH1 inhibition enriches activating H3K27ac at key glutathione biosynthetic genes. **a** Levels of oxidized glutathione (Y-axis) measured in chordoma cells treated with either IDH1 inhibitor or vehicle. (4 replicates each). **b** Diagram illustrating the key substrates (black) and proteins (green) involved in the glutathione biosynthesis. **c** Protein immunoblots for key glutathione pathway proteins SLC7A11, GCLM, and GSR with vinculin as loading control in three chordoma cell lines (UM-Chor1, CH22, and JHC7 from left to right) treated with vehicle or 3 µM IDH1 inhibitor for 48 h. **d** Scatter plots (Y-axis H3K27ac Log_2_ fold change, and X-axis Log_2_FC in gene expression) for three chordoma cell lines (UM-Chor1, CH22, and JHC7 from left to right) treated with vehicle or 3 µM of the IDH1 inhibitor for 48 h. Select NRF2 signature target genes are indicated by green enlarged points. **e** Motif analysis of H3K27ac sites that were commonly enriched across three chordoma cell lines (UM-Chor1, CH22, and JHC7). **f** Genome browser tracks showing H3K27ac signal (Y-axis) along the loci of three genes (*SLC7A11*, *GCLM*, and *GSR* from right to left) in the glutathione synthesis pathway (X-axis). Control (-) and IDH1 inhibitor-treated (+) tracks are shown for three cell lines: UM-Chor1 (green), CH22 (purple), and JHC7 (red). **g** and **h** Kaplan–Meier plots comparing probability (Y-axis) of overall survival over time in days (X-axis) for chordoma patients with either high (blue) or low (black) NRF2 gene signature. For g, n = 181, for h, n = 107, (h: data obtained from Zhang et al., 2024, Nature Communications)

NRF2 is a transcription factor that plays a critical role in regulating cellular redox response by driving expression of oxidative stress modulating genes. We queried 14 NRF2-driven gene signatures derived from 3600 lung cancer tumor samples with activated NRF2 signaling [11] (Table S12). 12 of these 14 genes, including *GCLM* (glutamate-cysteine ligase modifier subunit), cysteine-glutamate transporter *SLC7A11*, *GSR* (glutathione reductase), *FTH1* (ferritin heavy chain-1), *FTL1* (ferritin light chain-1), and *NQO1* [NAD(P)H:quinone oxidoreductase 1] were upregulated across all three cell lines on IDH1 inhibition (Fig. 3d).

Metabolomic analysis was conducted in parallel in each of these cell lines treated with IDH1i versus vehicle. We noted upregulation and downregulation of several metabolites (Fig. 3e) (Table S13). To assess functional significance, we performed an integrated pathway impact analysis by combining either upregulated (Table S14) or downregulated (Table S15) metabolite data with corresponding changes in gene expression (Fig. 3f). These data revealed upregulation of glutathione metabolism as the only common factor among all three cell lines (Fig. 3g). No common downregulated metabolic pathways were identified (Sup. Fig. S4f). These data led us to focus on glutathione in greater detail.

### IDH1 inhibition enriches activating H3K27ac at key glutathione biosynthetic genes

Data from the metabolomic assay show that levels of oxidized glutathione (GSSG) increased with IDH1 inhibitor treatment in all profiled cell lines (Fig. 4a). Intracellular glutathione exists in both oxidized (GSSG) and more labile reduced (GSH) forms. GSH detoxifies free radicals and, in this process, is converted to GSSG [5]. Glutathione is generated from cysteine, glycine, and glutamate via several well-studied steps mediated by NRF2 regulated proteins. SLC7A11 is a glutamate-cystine transporter, and GCLM links glutamate to cysteine in the glutathione biosynthetic pathway. GSR recycles GSSG to GSH (Fig. 4b). Our data suggest that chordoma cell lines respond to IDH1i by upregulating *SLC7A11*, *GCLM*, and *GSR* transcript levels. Accordingly, levels of all three proteins were also upregulated on IDH1i in multiple chordoma cells (Fig. 4c).

IDH1 inhibition lowered α-KG levels in all cell lines tested (Fig. 2h). Changes in α-KG levels can impact chromatin by altering post-translational methylation and acetylation marks on histone lysine residues. Histone demethylases belong to the αKG-dependent dioxygenase family and use α-KG as a cofactor for demethylation reactions [15, 34, 55]. Decreases in α-KG lower histone demethylase activity leading to an increase in histone methylation levels including H3K27me3 [6]. Conversely, recent studies have revealed that α-KG can regulate acetyl-CoA levels, which in turn is required for histone acetylation including H3K27ac deposition [27]. Furthermore, chordoma models have been shown to be sensitive to modulation of histone demethylase activity [10]. Based on this premise, it was necessary to evaluate if IDH1 inhibition would alter genomic distribution of both repressive H3K27me3 and activating H3K27ac marks corresponding with changes in gene expression.

We treated three chordoma cell lines with IDH1 inhibitor (3 μM, ~ IC_50_ levels) and performed chromatin immunoprecipitation followed by sequencing (ChIP-seq) studies for both repressive H3K27me3 and activating H3K27ac. We did not observe major changes in H3K27me3 and H3K27ac at the global level by immunoblotting (Sup. Fig. S5a). However, we noted changes in genomic distribution on IDH1 inhibition of both H3K27me3 and H3K27ac which varied among the three tested cell lines (Table S16–17). Genomic H3K27me3 was mainly enriched in non-promoter regions including at distal intergenic regions, simple repeats, introns, and gene bodies on IDH1 inhibition in all three cell lines to varying degrees (Sup. Figs. S5b–c). To determine functional significance on gene regulation, we also overlapped changes in gene expression with ChIP-seq data in all three cell lines. Minimal overlap between promoter H3K27me3 distribution and differences in gene expression was observed with IDH inhibitor treatment (Sup. Fig. 5d). In contrast, we noted both increased gene expression and genomic H3K27ac enrichment at several NRF2 signature targets including glutathione biosynthetic genes *SLC7A11*, *GCLM*, and *GSR* (Fig. 4d, f and Sup. Figs. S5e–f). Additionally, the motif analysis of differentially H3K27ac-enriched regions identified NRF2 along with other candidate binding transcription factors (Fig. 4e). These data are consistent with the ability of NRF2 to enrich for H3K27ac and thereby activate expression of its target genes [30]. Together, our findings suggest that IDH1 inhibition activates NRF2 to increase both H3K27ac enrichment and gene expression of key glutathione biosynthetic genes corresponding to an increase in GSSG levels. We also observed poor overall survival in chordoma patients with high median expression of the 14-gene NRF2 signature gene set (Table S12, Fig. 4g, h) leading us to further explore glutathione-dependent antioxidant metabolism.

**Fig. 5:**
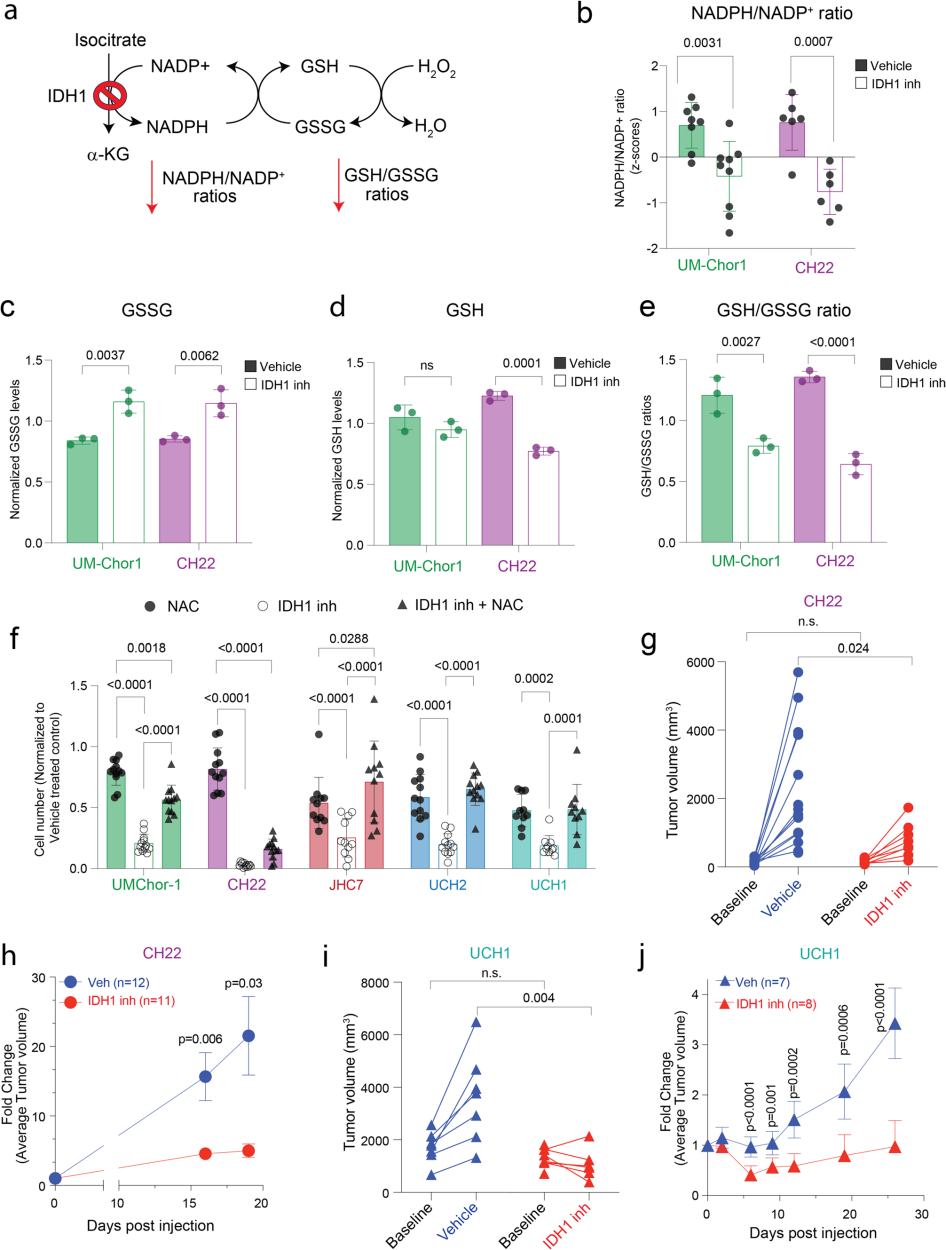
IDH1 inhibition lowers NADPH/NADP^+^ and GSH/GSSG ratios and toxicity is attenuated with antioxidant supplementation. **a** Schematic illustrating the reaction catalyzed by IDH1 in the setting of redox regulation. IDH1 converts NADP+ to NADPH when it metabolizes isocitrate to α-KG. NADPH is required by GSH to reduce oxidative molecules such as H_2_O_2_. GSH is converted to GSSG during this reaction. Hypothesized reduction of both NADPH/NADP+ and GSH/GSSG ratios on IDH1 inhibition are indicated by the red arrows. **b** Z-scores of NADPH/NADP^+^ ratios (Y-axis) measured in two indicated chordoma cells treated with IDH1 inhibitor (no fill, 3 µM for 48 h) or vehicle (UM-Chor1 in green and CH22 in purple fill, DMSO). Data for UM-Chor1 were generated from two separate experiments of four technical replicates each. Data for CH22 were generated from two separate experiments of three technical replicates each. **c** Levels of oxidized (GSSG) glutathione (Y-axis, a.u.) across two indicated cell lines treated with IDH1 inhibitor (no fill, 3 µM for 48 h) or vehicle (solid fill, DMSO). **d** Levels of reduced (GSH) glutathione (Y-axis, a.u.) across two indicated cell lines treated with IDH1 inhibitor (no fill, 3 µM for 48 h) or vehicle (solid fill, DMSO). **e** Ratios of reduced GSH/GSSG (Y-axis, a.u.) across two indicated cell lines treated with IDH1 inhibitor (no fill, 3 µM for 48 h) or vehicle (solid fill, DMSO). (*n* = 3 experiments; GSH/GSSG was calculated independently for each condition and then normalized to the vehicle condition). **f** Cell counts normalized to a double-vehicle (DMDO and PBS) control (Y-axis, a.u.) in five indicated chordoma cell-line models treated with either 5 mM N-acetylcysteine (NAC), 5 µM IDH1 inhibitor or a combination of the compounds and counted after four days (X-axis). Six experiments with 2–3 replicates per experiment. **g** Tumor volumes for vehicle (DMSO, blue, *n* = 12) or IDH1 inhibitor-treated (red, *n* = 11) CH22 flank xenografts at baseline versus end of trial (20 days post injection). **h** Tumor growth curves measuring fold change in volume from pre-treatment baseline measurements (Y-axis) over time from the start of treatment in days (X-axis) comparing vehicle (DMSO, blue, *n* = 12) or IDH1 inhibitor-treated (red, *n* = 11) CH22 flank xenografts. **i** Tumor volumes for vehicle (DMSO, blue, n = 7) or IDH1 inhibitor-treated (red, *n* = 8) U-CH1 flank xenografts at baseline versus end of trial (26 days post injection). **j** Tumor growth curves measuring fold change in volume from pre-treatment baseline measurements (Y-axis) over time from the start of treatment in days (X-axis) comparing vehicle (DMSO, blue, *n* = 7) or IDH1 inhibitor-treated (red, *n* = 8) U-CH1 flank xenografts. Data are plotted as means ± SD. Data analyzed in b-d analyzed by 2-sided, unpaired, 2-tailed Student’s t test; in f by ANOVA, and in g–j by Mann–Whitney U-test; all with 95% confidence intervals

### IDH1 inhibition lowers NADPH/NADP^+^ and GSH/GSSG ratios and toxicity is attenuated with antioxidant supplementation

Glutathione metabolism is driven by NADPH/NADP^+^ ratios with high ratios promoting recycling of glutathione from GSSG to GSH, which in turn detoxifies free radicals [48] (Fig. 5a). GSH/GSSG ratios are a critical indicator of intracellular redox response capacity and low GSH/GSSG ratios can lead to oxidative damage and cell death [17]. NADPH is a product of the cytosolic reaction catalyzed by IDH1 and its generation is critical for glutathione recycling [16]. Therefore, we hypothesized that IDH1 inhibitor lowers NADPH/NADP^+^ to disrupt GSSG recycling resulting in decreased GSH/GSSG ratios (Fig. 5a). To assess this proposed model, we measured both NADPH/NADP+ ratios in chordoma cell lines and found that they dropped with IDH1 inhibitor treatment (Fig. 5b). GSH is a highly labile metabolite and is challenging to accurately measure by our global metabolomic assays [26]. We therefore applied a commercially developed assay to accurately assess the balance of GSSG and GSH pools in chordoma cells. GSSG levels rose with treatment, consistent with metabolomics data (Fig. 5c). While GSH levels only significantly dropped in one of the two cell lines tested (Fig. 5d), both cell lines exhibited a significant reduction in the GSH/GSSG ratio on IDH1 inhibition (Fig. 5e).

Considering these findings, we hypothesized that amelioration of oxidative stress could reduce the toxic effects of IDH1 inhibition. *N*-Acetylcysteine is an antioxidant that serves as a backbone for glutathione production. While mildly toxic to cells on its own (Fig. 5f, first column for each cell line), *N*-acetylcysteine supplementation partially (UM-Chor1, CH22) or fully (JCH7, U-CH2, U-CH1) rescued proliferation defects with IDH1i treatment across multiple chordoma cell lines (Fig. 5f). Treatment of UM-Chor1, CH22, and JHC7 with oxalomalate, a wild-type IDH1 and IDH2 inhibitor with less specificity [19, 20, 50, 56], resulted in proliferation inhibition (Sup. Fig. 5g) at similar doses to previously characterized melanoma models [19]. We observed increased protein levels of SLC7A11, GCLM, and GSR in UM-Chor1 and JCH7 cells (Sup. Fig. 5h). Together, these data demonstrate that treatment with an IDH1-targeting inhibitor lowers both NADPH/NADP^+^ and GSH/GSSG ratios, and toxicity is rescued by NAC supplementation.

### IDH1 inhibition is therapeutic in vivo

We examined whether the observed toxicity effects of IDH1 inhibition in cell culture would also be observed in vivo. Using the CH22 chordoma cells in NOD scid gamma (NSG) immunodeficient mice, we generated flank tumors and randomized animals to IDH1i treatment or vehicle control. The volume of tumors at the study endpoint was significantly decreased in vehicle versus IDH1i-treated animals (Fig. 5g) and the tumor fold change was lower at longitudinal timepoints within the study (Fig. 5h). We validated our findings in an additional U-CH1 xenograft model. Tumor volume at endpoint and fold change growth at serial time points throughout the treatment course were significantly lower in mice treated with the IDH1i compared to vehicle-treated control animals (Fig. 5i–j). Tumor weights at endpoint were also decreased with IDH1i treatment for both models studied (Sup. Figs. 6a–c), additionally, immunohistochemistry was performed on the extracted tumors to validate the IDH1 expression in the subcutaneous tumors (Sup. Figs. 6d).

## Discussion

Chordomas are aggressive and difficult-to-treat tumors, and we sought to determine metabolic targets to uncover potential therapeutic strategies. We identify IDH1 upregulation and demonstrate that its elevated expression is associated with worse survival in a cohort of chordoma patients. Targeting IDH1 via shRNA and siRNA, or pharmacologic inhibition in chordoma cells was toxic in vitro. Data from transcriptomics, regulatory chromatin profiles, and metabolomic assays converged on upregulation of glutathione synthesis machinery. Specifically, we noted increased H3K27ac deposition at NRF2 regulatory genes corresponding to upregulation of key glutathione biosynthetic genes as the primary response of chordoma cells to this inhibitor. GSSG levels were increased, and GSH/GSSG ratios were lower in IDH1i versus vehicle-treated cells. This was accompanied by reduced NADPH/NADP^+^ ratios, which drive reduction of GSSG to GSH. In support of these findings, N-acetylcysteine rescued chordoma cell proliferation deficits when treated with IDH1i. Finally, we demonstrate the efficacy of an IDH1-targeting inhibitor in vivo.

IDH1 mutations (IDH1m) impact both epigenetic and metabolic pathways. IDH1m produces D-2HG which is structurally similar to α-KG and competitively inhibits histone demethylases to increase histone lysine methylation marks including H3K27me3. Moreover, the reaction producing D-2HG consumes NADPH [3] Multiple lines of evidence show the potential of targeting glutathione dependences in IDH1m tumor models [42, 47, 52, 53]. Our data suggests that inhibition of wild type IDH1 engages similar pathways by activating NRF2 signatures in chordoma cells. However, in contrast to IDH1m, changes in H3K27me3 genomic distribution did not correspond to alterations in gene expression and were mainly observed in non-promoter regions (simple repeats, introns, gene bodies and distal intergenic regions) on IDH1 inhibition. NRF2 has been shown to reprogram genomic distribution of H3K27ac and enhancer landscapes [30] We observed a similar phenotype with H3K27ac enrichment at several NRF2 target genes on IDH1 inhibition in multiple chordoma models.

Recent work has elucidated that an immune-mediated signaling axis promotes increased cholesterol metabolism in chordoma tumors that in turn is associated with increased stemness and features of tumor progression [57]. Intriguingly, *IDH1* is a known transcriptional target of sterol regulatory element-binding proteins (SREBPs), key transcription factors involved in lipogenesis, and mutations in *IDH1* disrupt lipid synthesis [39, 43]. Sterol regulatory element-binding transcription factor 1 (*SREBF1*), the gene that encodes SREBP-1, is significantly differentially expressed in *IDH1*-high populations compared to *IDH1-*low populations in the available scRNA-seq. Additional work will be needed to interrogate whether SREBP-1 directly regulates *IDH1* expression in chordoma, and whether heterogeneity in cholesterol metabolism mirrors heterogeneity observed in *IDH1* expression among chordoma cells. Moreover, studies in both pediatric and adult chordomas have identified heteroplasmic mitochondrial DNA mutations in key NADH complex genes as well as a dependency on expression of superoxide dismutases 1 and 2 [29, 37]. Mutations were observed in mitochondrial complex 1 genes encoding NADH complex including *MT-ND5*, *MT-ND1*, and *MT-ND4*, implying that mitochondrial metabolic pathways that regulate redox may be a key vulnerability. In the setting of potential selection for disruption of NADH homeostasis in chordoma development, chordoma cell reliance on alternative pools of reducing agents through superoxide dismutases or upregulation of the NADPH-producing IDH1 may be heightened. The study detailed here provides further evidence of potential redox-related susceptibilities using a compound that targets IDH1 in vitro and in vivo efficacy in chordoma that warrant further investigation.

## Supplementary Information

Below is the link to the electronic supplementary material.

Supplementary file1 (PDF 1741 KB)

## Data Availability

RNA-sequencing and ChIP-sequencing data generated for this study have been deposited in the Gene Expression Omnibus (GEO) with accession number GSE334701. Results from these experiments as well as metabolomic results are included in supplementary files for this study. Previously published datasets utilized for analyses in this study were accessed as described in the Methods section above.

## References

[1] Amary MF, Bacsi K, Maggiani F, Damato S, Halai D, Berisha F et al (2011) *IDH1* and *IDH2* mutations are frequent events in central chondrosarcoma and central and periosteal chondromas but not in other mesenchymal tumours. J Pathol 224:334–343 21598255 10.1002/path.2913

[2] Amemiya HM, Kundaje A, Boyle AP (2019) The ENCODE blacklist: identification of problematic regions of the genome. Sci Rep 9:9354. 10.1038/s41598-019-45839-z 31249361 10.1038/s41598-019-45839-z PMC6597582

[3] Biedermann J, Preussler M, Conde M, Peitzsch M, Richter S, Wiedemuth R et al (2019) Mutant *IDH1* differently affects redox state and metabolism in glial cells of normal and tumor origin. Cancers (Basel). 10.3390/cancers11122028 31888244 10.3390/cancers11122028 PMC6966450

[4] Bray NL, Pimentel H, Melsted P, Pachter L (2016) Near-optimal probabilistic RNA-seq quantification. Nat Biotechnol 34:525–527. 10.1038/nbt.3519 27043002 10.1038/nbt.3519

[5] Browne RW, Armstrong D (1998) Reduced glutathione and glutathione disulfide. Methods Mol Biol 108:347–352. 10.1385/0-89603-472-0:347 9921543 10.1385/0-89603-472-0:347

[6] Carey BW, Finley LWS, Cross JR, Allis CD, Thompson CB (2015) Intracellular α-ketoglutarate maintains the pluripotency of embryonic stem cells. Nature 518:413–416. 10.1038/nature13981 25487152 10.1038/nature13981 PMC4336218

[7] Chen EY, Tan CM, Kou Y, Duan Q, Wang Z, Meirelles GV et al (2013) Enrichr: interactive and collaborative HTML5 gene list enrichment analysis tool. BMC Bioinformatics 14:128. 10.1186/1471-2105-14-128 23586463 10.1186/1471-2105-14-128 PMC3637064

[8] Chen S, Xu W, Jiao J, Jiang D, Liu J, Chen T et al (2015) Differential proteomic profiling of primary and recurrent chordomas. Oncol Rep 33:2207–2218 25738923 10.3892/or.2015.3818

[9] Chung C, Sweha SR, Pratt D, Tamrazi B, Panwalkar P, Banda A et al (2020) Integrated metabolic and epigenomic reprograming by H3K27M mutations in diffuse intrinsic pontine gliomas. Cancer Cell 38:334-349.e9. 10.1016/j.ccell.2020.07.008 32795401 10.1016/j.ccell.2020.07.008 PMC7494613

[10] Cottone L, Cribbs AP, Khandelwal G, Wells G, Ligammari L, Philpott M et al (2020) Inhibition of histone H3K27 demethylases inactivates brachyury (TBXT) and promotes chordoma cell death. Cancer Res 80:4540–4551. 10.1158/0008-5472.CAN-20-1387 32855205 10.1158/0008-5472.CAN-20-1387 PMC7616956

[11] Crippa V, Cordani N, Villa AM, Malighetti F, Villa M, Sala L et al (2025) Integrative analysis of KEAP1/NFE2L2 alterations across 3600+ tumors reveals an NRF2 expression signature as a prognostic biomarker in cancer. npj Precis Oncol 9:291. 10.1038/s41698-025-01088-0 40825842 10.1038/s41698-025-01088-0 PMC12361507

[12] Dahlin DC, Maccarty CS (1952) Chordoma. A study of fifty‐nine cases. Cancer 5:1170–1178 12998023 10.1002/1097-0142(195211)5:6<1170::aid-cncr2820050613>3.0.co;2-c

[13] Halbrook CJ, Pontious C, Kovalenko I, Lapienyte L, Dreyer S, Lee H-J et al (2019) Macrophage-released pyrimidines inhibit gemcitabine therapy in pancreatic cancer. Cell Metab 29:1390-1399.e6. 10.1016/j.cmet.2019.02.001 30827862 10.1016/j.cmet.2019.02.001 PMC6602533

[14] Hsu W, Mohyeldin A, Shah SR, ap Rhys CM, Johnson LF, Sedora-Roman NI et al (2011) Generation of chordoma cell line JHC7 and the identification of Brachyury as a novel molecular target. J Neurosurg 115:760–769. 10.3171/2011.5.JNS11185 21699479 10.3171/2011.5.JNS11185 PMC4273567

[15] Hutton JJ Jr, Tappel AL, Udenfriend S (1967) Cofactor and substrate requirements of collagen proline hydroxylase. Arch Biochem Biophys 118:231–240

[16] Jo S-H, Son M-K, Koh H-J, Lee S-M, Song I-H, Kim Y-O et al (2001) Control of mitochondrial redox balance and cellular defense against oxidative damage by mitochondrial NADP+-dependent isocitrate dehydrogenase*. J Biol Chem 276:16168–16176. 10.1074/jbc.M010120200 11278619 10.1074/jbc.M010120200

[17] Kalinina EV, Gavriliuk LA (2020) Glutathione synthesis in cancer cells. Biochemistry (Mosc) 85:895–907. 10.1134/S0006297920080052 33045950 10.1134/S0006297920080052

[18] Kaneva K, O’Halloran K, Triska P, Liu X, Merkurjev D, Bootwalla M et al (2021) The spectrum of mitochondrial DNA (mtDNA) mutations in pediatric CNS tumors. Neurooncol Adv 3:vdab074 34337412 10.1093/noajnl/vdab074 PMC8320689

[19] Kim SH, Kil IS, Kwon O-S, Kang BS, Lee D-S, Lee H-S et al (2019) Oxalomalate reduces tumor progression in melanoma via ROS-dependent proapoptotic and antiangiogenic effects. Biochimie 158:165–171. 10.1016/j.biochi.2019.01.004 30639360 10.1016/j.biochi.2019.01.004

[20] Kim SH, Kim H, Lee JH, Park J-W (2019) Oxalomalate suppresses metastatic melanoma through IDH-targeted stress response to ROS. Free Radic Res 53:418–429. 10.1080/10715762.2019.1597974 31020875 10.1080/10715762.2019.1597974

[21] Kuleshov MV, Jones MR, Rouillard AD, Fernandez NF, Duan Q, Wang Z et al (2016) Enrichr: a comprehensive gene set enrichment analysis web server 2016 update. Nucleic Acids Res 44:w90–w97. 10.1093/nar/gkw377 27141961 10.1093/nar/gkw377 PMC4987924

[22] Lee H-J, Kremer DM, Sajjakulnukit P, Zhang L, Lyssiotis CA (2019) A large-scale analysis of targeted metabolomics data from heterogeneous biological samples provides insights into metabolite dynamics. Metabolomics 15:1–13 10.1007/s11306-019-1564-8 PMC6616221 31289941

[23] Love MI, Huber W, Anders S (2014) Moderated estimation of fold change and dispersion for RNA-seq data with DESeq2. Genome Biol 15:550. 10.1186/s13059-014-0550-8 25516281 10.1186/s13059-014-0550-8 PMC4302049

[24] Mardis ER, Ding L, Dooling DJ, Larson DE, McLellan MD, Chen K et al (2009) Recurring mutations found by sequencing an acute myeloid leukemia genome. N Engl J Med 361:1058–1066. 10.1056/NEJMoa0903840 19657110 10.1056/NEJMoa0903840 PMC3201812

[25] McMaster ML, Goldstein AM, Bromley CM, Ishibe N, Parry DM (2001) Chordoma: incidence and survival patterns in the United States, 1973–1995. Cancer Causes Control 12:1–11 11227920 10.1023/a:1008947301735

[26] Nagana Gowda GA, Pascua V, Raftery D (2021) Extending the scope of (1)H NMR-based blood metabolomics for the analysis of labile antioxidants: reduced and oxidized glutathione. Anal Chem 93:14844–14850. 10.1021/acs.analchem.1c03763 34704738 10.1021/acs.analchem.1c03763 PMC8822164

[27] Van Nerum K, Wenzel A, Argemi-Muntadas L, Kafkia E, Drews A, Brun IS et al (2025) α-Ketoglutarate promotes trophectoderm induction and maturation from naive human embryonic stem cells. Nat Cell Biol 27:749–761. 10.1038/s41556-025-01658-1 40269259 10.1038/s41556-025-01658-1 PMC12081308

[28] O’Halloran K, Hakimjavadi H, Bootwalla M, Ostrow D, Kerawala R, Cotter JA et al (2024) Pediatric chordoma: a tale of two genomes. Mol Cancer Res 22:721–729. 10.1158/1541-7786.MCR-23-0741 38691518 10.1158/1541-7786.MCR-23-0741 PMC11296893

[29] O’Halloran K, Hakimjavadi H, Bootwalla M, Ostrow D, Kerawala R, Cotter JA et al (2024) Pediatric Chordoma: A Tale of Two Genomes. Molecul Cancer Res 22(8):721–729 10.1158/1541-7786.MCR-23-0741 PMC11296893 38691518

[30] Okazaki K, Anzawa H, Liu Z, Ota N, Kitamura H, Onodera Y et al (2020) Enhancer remodeling promotes tumor-initiating activity in NRF2-activated non-small cell lung cancers. Nat Commun 11:5911. 10.1038/s41467-020-19593-0 33219226 10.1038/s41467-020-19593-0 PMC7679411

[31] Olson JT, Wenger DE, Rose PS, Petersen IA, Broski SM (2021) Chordoma: 18F-FDG PET/CT and MRI imaging features. Skeletal Radiol 50:1657–1666. 10.1007/s00256-021-03723-w 33521875 10.1007/s00256-021-03723-w

[32] Ostroumov E, Hunter CJ (2007) The role of extracellular factors in human metastatic chordoma cell growth in vitro. Spine (Phila Pa 1976) 32:2957–2964. 10.1097/BRS.0b013e31815cde91 18091487 10.1097/BRS.0b013e31815cde91

[33] Parsons DW, Jones S, Zhang X, Lin JC-H, Leary RJ, Angenendt P et al (2008) An integrated genomic analysis of human glioblastoma multiforme. Science 321:1807–1812 18772396 10.1126/science.1164382 PMC2820389

[34] Prescott AG, John P (1996) Dioxygenases: molecular structure and role in plant metabolism. Annu Rev Plant Physiol Plant Mol Biol 47:245–271. 10.1146/annurev.arplant.47.1.245 15012289 10.1146/annurev.arplant.47.1.245

[35] Presneau N, Shalaby A, Ye H, Pillay N, Halai D, Idowu B et al (2011) Role of the transcription factor T (brachyury) in the pathogenesis of sporadic chordoma: a genetic and functional-based study. J Pathol 223:327–335. 10.1002/path.2816 21171078 10.1002/path.2816

[36] Ramírez F, Ryan DP, Grüning B, Bhardwaj V, Kilpert F, Richter AS et al (2016) DeepTools2: a next generation web server for deep-sequencing data analysis. Nucleic Acids Res 44:W160. 10.1093/nar/gkw257 27079975 10.1093/nar/gkw257 PMC4987876

[37] Seven D, Tecimel D, Özbey U, Kızılilsoley N, Nikerel E, Dalan AB et al (2025) Silencing superoxide dismutases (SOD1&SOD2) potentiates ROS-induced apoptosis in chordoma cells. Mol Biol Rep 52:157 39853601 10.1007/s11033-025-10239-2

[38] Sharifnia T, Wawer MJ, Chen T, Huang Q-Y, Weir BA, Sizemore A et al (2019) Small-molecule targeting of brachyury transcription factor addiction in chordoma. Nat Med 25:292–300. 10.1038/s41591-018-0312-3 30664779 10.1038/s41591-018-0312-3 PMC6633917

[39] Shechter I, Dai P, Huo L, Guan G (2003) IDH1 gene transcription is sterol regulated and activated by SREBP-1a and SREBP-2 in human hepatoma HepG2 cells: evidence that IDH1 may regulate lipogenesis in hepatic cells. J Lipid Res 44:2169–2180. 10.1194/jlr.M300285-JLR200 12923220 10.1194/jlr.M300285-JLR200

[40] Shen Y, Li M, Xiong Y, Gui S, Bai J, Zhang Y et al (2021) Proteomics analysis identified ASNS as a novel biomarker for predicting recurrence of skull base chordoma. Front Oncol 11:698497. 10.3389/fonc.2021.698497 34540668 10.3389/fonc.2021.698497 PMC8440958

[41] Sheppard HE, Dall’Agnese A, Park WD, Shamim MH, Dubrulle J, Johnson HL et al (2021) Targeted brachyury degradation disrupts a highly specific autoregulatory program controlling chordoma cell identity. Cell Rep Med 2:100188. 10.1016/j.xcrm.2020.100188 33521702 10.1016/j.xcrm.2020.100188 PMC7817874

[42] Tang X, Fu X, Liu Y, Yu D, Cai SJ, Yang C (2020) Blockade of glutathione metabolism in IDH1-mutated glioma. Mol Cancer Ther 19:221–230. 10.1158/1535-7163.MCT-19-0103 31548295 10.1158/1535-7163.MCT-19-0103 PMC6946871

[43] Thomas D, Wu M, Nakauchi Y, Zheng M, Thompson-Peach CAL, Lim K et al (2023) Dysregulated lipid synthesis by oncogenic IDH1 mutation is a targetable synthetic lethal vulnerability. Cancer Discov 13:496–515. 10.1158/2159-8290.CD-21-0218 36355448 10.1158/2159-8290.CD-21-0218 PMC9900324

[44] Vaziri-Gohar A, Cassel J, Mohammed FS, Zarei M, Hue JJ, Hajihassani O et al (2022) Limited nutrient availability in the tumor microenvironment renders pancreatic tumors sensitive to allosteric IDH1 inhibitors. Nat Cancer 3:852–865 35681100 10.1038/s43018-022-00393-y PMC9325670

[45] Vujovic S, Henderson S, Presneau N, Odell E, Jacques TS, Tirabosco R et al (2006) Brachyury, a crucial regulator of notochordal development, is a novel biomarker for chordomas. J Pathol 209:157–165 16538613 10.1002/path.1969

[46] Walcott BP, Nahed BV, Mohyeldin A, Coumans J-V, Kahle KT, Ferreira MJ (2012) Chordoma: current concepts, management, and future directions. Lancet Oncol 13:e69-76. 10.1016/S1470-2045(11)70337-0 22300861 10.1016/S1470-2045(11)70337-0

[47] Wang T-X, Liang J-Y, Zhang C, Xiong Y, Guan K-L, Yuan H-X (2019) The oncometabolite 2-hydroxyglutarate produced by mutant IDH1 sensitizes cells to ferroptosis. Cell Death Dis 10:755. 10.1038/s41419-019-1984-4 31591388 10.1038/s41419-019-1984-4 PMC6779886

[48] Xiao W, Loscalzo J (2020) Metabolic responses to reductive stress. Antioxid Redox Signal 32:1330–1347. 10.1089/ars.2019.7803 31218894 10.1089/ars.2019.7803 PMC7247050

[49] Xie Z, Bailey A, Kuleshov MV, Clarke DJB, Evangelista JE, Jenkins SL et al (2021) Gene Set Knowledge Discovery with Enrichr. Curr Protoc 1:90. 10.1002/cpz1.90 10.1002/cpz1.90 PMC8152575 33780170

[50] Xu W, Yang H, Liu Y, Yang Y, Wang P, Kim S-H et al (2011) Oncometabolite 2-hydroxyglutarate is a competitive inhibitor of α-ketoglutarate-dependent dioxygenases. Cancer Cell 19:17–30. 10.1016/j.ccr.2010.12.014 21251613 10.1016/j.ccr.2010.12.014 PMC3229304

[51] Yang C, Hornicek FJ, Wood KB, Schwab JH, Choy E, Iafrate J et al (2010) Characterization and analysis of human chordoma cell lines. Spine (Phila Pa 1976) 35:1257–1264. 10.1097/BRS.0b013e3181c2a8b0 20461036 10.1097/BRS.0b013e3181c2a8b0 PMC3769690

[52] Yu D, Liu Y, Zhou Y, Ruiz-Rodado V, Larion M, Xu G et al (2020) Triptolide suppresses IDH1-mutated malignancy via Nrf2-driven glutathione metabolism. Proc Natl Acad Sci U S A 117:9964–9972. 10.1073/pnas.1913633117 32312817 10.1073/pnas.1913633117 PMC7211987

[53] Zarei M, Hajihassani O, Hue JJ, Graor HJ, Loftus AW, Rathore M et al (2022) Wild-type IDH1 inhibition enhances chemotherapy response in melanoma. J Exp Clin Cancer Res 41:1–18 36153582 10.1186/s13046-022-02489-w PMC9509573

[54] Zarei M, Hue JJ, Hajihassani O, Graor HJ, Katayama ES, Loftus AW et al (2021) Clinical development of IDH1 inhibitors for cancer therapy. Cancer Treat Rev. 10.1016/j.ctrv.2021.102334 34974243 10.1016/j.ctrv.2021.102334

[55] Zdzisińska B, Żurek A, Kandefer-Szerszeń M (2017) Alpha-ketoglutarate as a molecule with pleiotropic activity: well-known and novel possibilities of therapeutic use. Arch Immunol Ther Exp (Warsz) 65:21–36. 10.1007/s00005-016-0406-x 27326424 10.1007/s00005-016-0406-x PMC5274648

[56] Zhao S, Lin Y, Xu W, Jiang W, Zha Z, Wang P et al (1979) Xiong Y (2009) Glioma-Derived Mutations in IDH1 Dominantly Inhibit IDH1 Catalytic Activity and Induce HIF-1α. Science 324:261–265. 10.1126/science.1170944 10.1126/science.1170944 PMC3251015 19359588

[57] Zheng B-W, Xia C, Huang W, Niu H-Q, Luo B-M, Liang S-Q et al (2026) Cholesterol-metabolic tumor-associated macrophages regulate tumor budding-like cell subpopulation to promote chordoma stemness via BACH1/ANGPTL4/SDC4 axis. Neuro Oncol 28:675–689. 10.1093/neuonc/noaf286 41390963 10.1093/neuonc/noaf286 PMC13070493

