# Supplementary material for: Targeting chordoma via an isocitrate dehydrogenase-1-dependent susceptibility to redox metabolism
Source: Acta Neuropathol. 2026 Jul 23;152(1):10. doi: 10.1007/s00401-026-03048-9 (PMC13396039; doi:10.1007/s00401-026-03048-9)
Supplement: Supplementary file 1 — Supplementary file1 (PDF 1741 KB) [file 401_2026_3048_MOESM1_ESM.pdf]

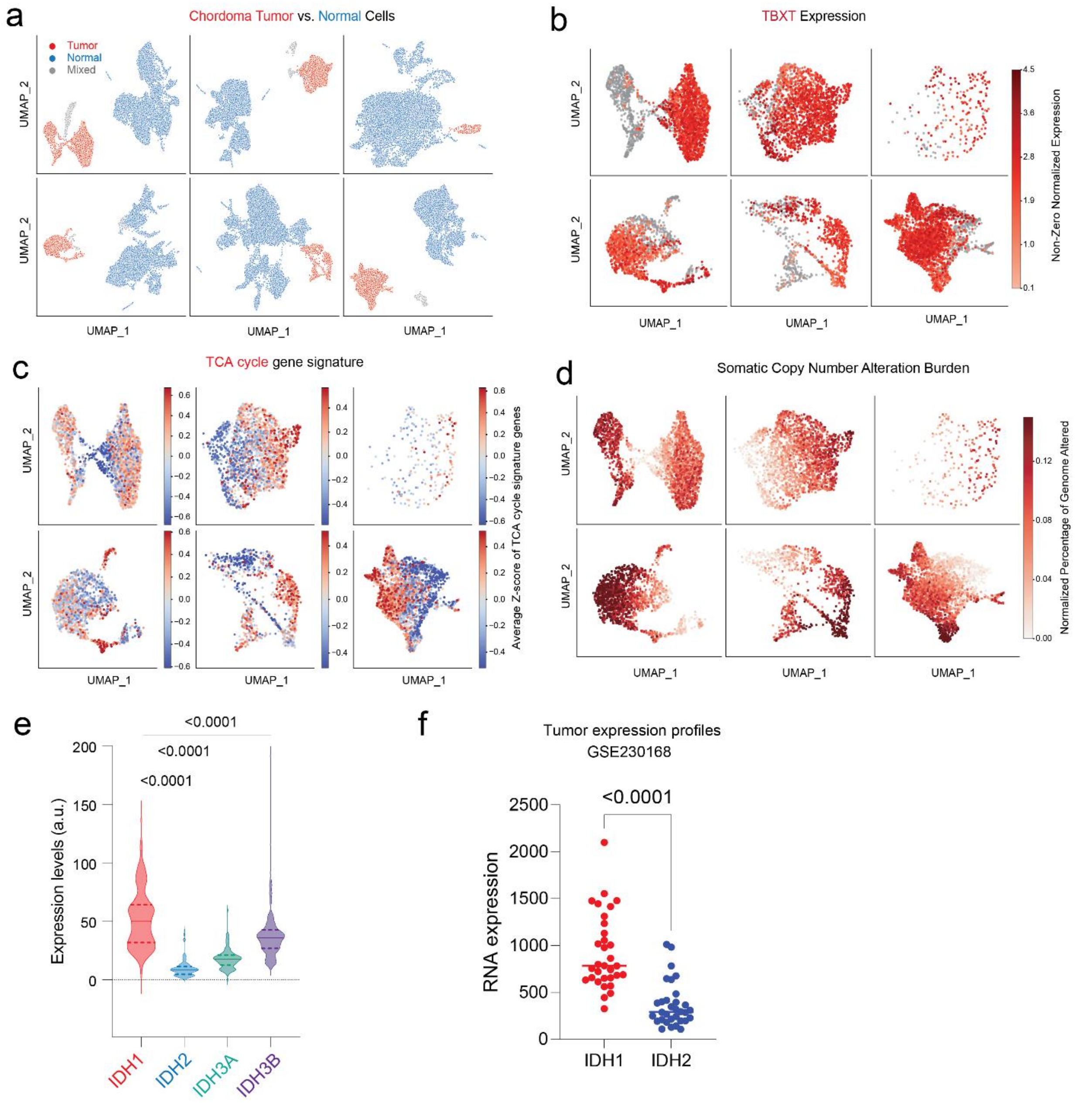

Supplementary Fig. 1 <u>*IDH1* expression is elevated in chordomas and is associated with poor clinical outcomes</u>

a) UMAP projections of all single cells from six chordoma tumors colored as neoplastic, non-neoplastic, or mixed based on TBXT expression and SCNA profiles. Tumor and normal populations form clearly separable clusters.

b) UMAP projections of neoplastic cells colored by log-normalized TBXT expression, showing its high prevalence and spatial heterogeneity across tumors. Grey points represent TBXT-negative cells.

c) UMAP projections of neoplastic cells from six chordoma tumors colored by a 27-gene TCA-cycle metabolic signature score. Warmer colors mark regions with higher metabolic activity.

d) UMAP projections of neoplastic cells from six chordoma tumors colored based on somatic copy number alteration burden. Warmer colors mark regions with higher somatic copy number alteration.

e) Comparison of the spread and average expression levels of *IDH1, IDH2, IDH3A, and IDH3B* (expression levels, a.u., Y-axis) in chordoma samples (n=35, X-axis) using the same dataset from Fig. 1b.

f) Comparison of the expression of *IDH1* and *IDH2* in chordoma tumor tissues from publicly available data set, GSE230168.

Data are plotted as mean ± SD. Data in e analyzed by ANOVA and in f by unpaired Student's t-test with 95% c.i.

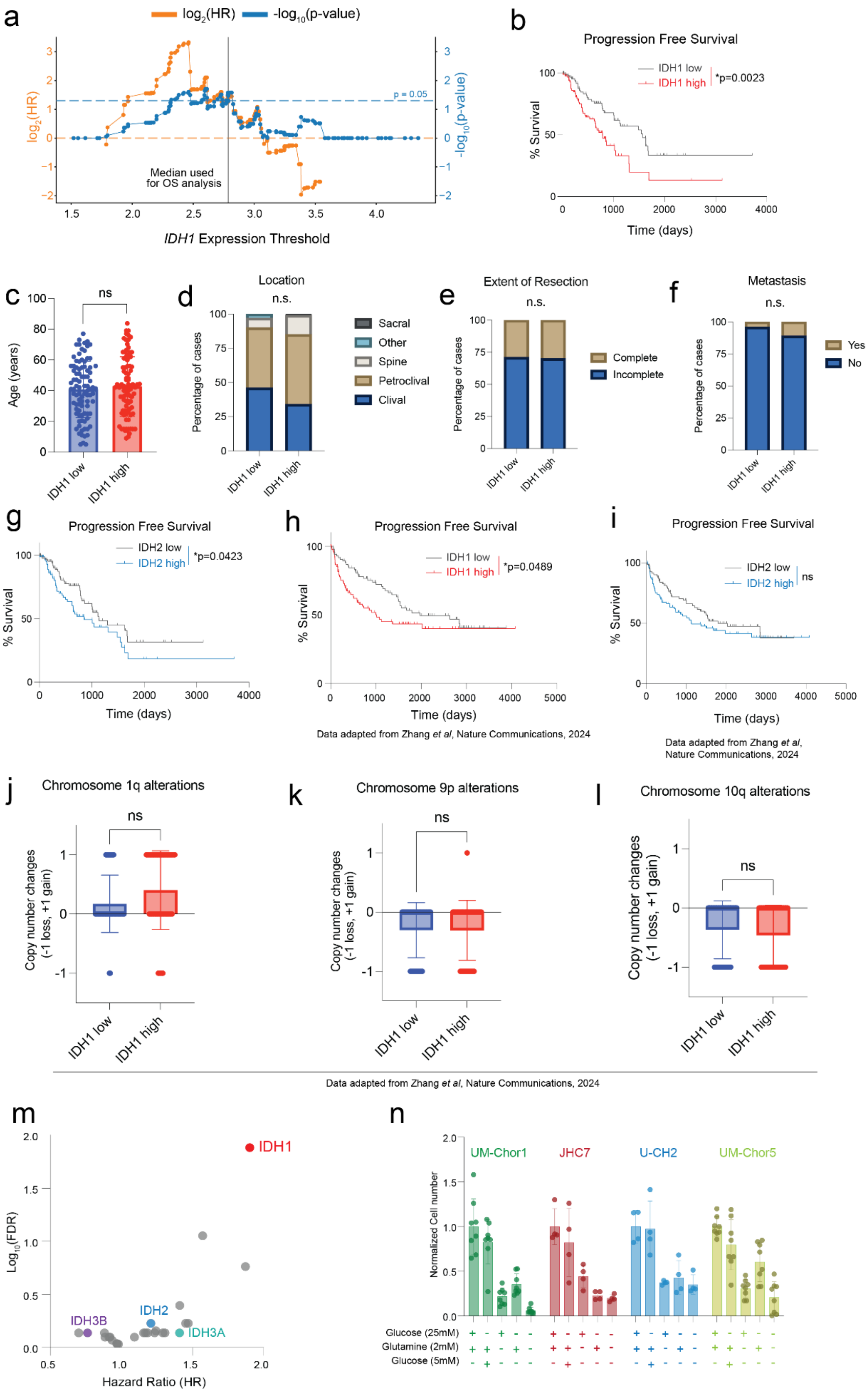
a
log2(HR)
-log10(p-value)
p = 0.05
Median used for OS analysis
IDH1 Expression Threshold
b
Progression Free Survival
IDH1 low
IDH1 high
*p=0.0023
% Survival
Time (days)
c
ns
Age (years)
IDH1 low
IDH1 high
d
Location
n.s.
Percentage of cases
Sacral
Other
Spine
Petroclival
Clival
e
Extent of Resection
n.s.
Complete
Incomplete
f
Metastasis
n.s.
Yes
No
g
Progression Free Survival
IDH2 low
IDH2 high
*p=0.0423
h
Progression Free Survival
IDH1 low
IDH1 high
*p=0.0489
Data adapted from Zhang et al, Nature Communications, 2024
i
Progression Free Survival
IDH2 low
IDH2 high
ns
Data adapted from Zhang et al, Nature Communications, 2024
j
Chromosome 1q alterations
k
Chromosome 9p alterations
l
Chromosome 10q alterations
Copy number changes (-1 loss, +1 gain)
Data adapted from Zhang et al, Nature Communications, 2024
m
IDH1
IDH2
IDH3B
IDH3A
Log10(FDR)
Hazard Ratio (HR)
n
UM-Chor1
JHC7
U-CH2
UM-Chor5
Normalized Cell number
Glucose (25mM)
Glutamine (2mM)
Glucose (5mM)

Supplementary Fig. 2 *IDH1* expression is elevated in chordomas and associated with poor clinical outcomes

a) Correlation between survival (Y- axis) and *IDH1* gene expression (X-axis). Two halves separated by line representing median *IDH1* expression.

b) Kaplan-Meier plot comparing probability of progression free survival (Y-axis) over time in days (X-axis) for chordoma patients with either high (blue, above median) or low (purple, below median) expression of *IDH1* in a cohort of skull-base chordoma patients (n=181).

c – f) Distribution of IDH1 high vs IDH1 low Chordoma tumors w.r.t. age (c), location (d), extent of resection (e) and metastasis (f).

g) Kaplan-Meier plot comparing probability of progression free survival (Y-axis) over time in days (X-axis) for chordoma patients with either high (blue, above median) or low (purple, below median) expression of *IDH2* in a cohort of skull-base chordoma patients (n=181).

h and i) Kaplan-Meier plot comparing probability (Y-axis) of progression free survival over time in days (X-axis) for chordoma patients with either high (blue) or low (purple) expression of *IDH1* (h) and *IDH2* (i) using data obtained from Zhang et al. (2024), stratified by median expression level (n=104).

j-l) Distribution of commonly found chromatin alterations; 1q (i), 9p (j), 10q (k), in chordoma between IDH1 high vs IDH1 low Chordoma tumors

m) Volcano plot representing cox proportional hazards models for progression free survival using for all TCA cycle related genes (IDH1, p = 0.0005, FDR = 0.013).

n) Cell counts (Y-axis) of chordoma cell lines (denoted with distinct colors) demonstrating sensitivity to withdrawal of glucose, or glutamine, or both (Cell counts normalized to full media control, X-axis) (n=3 separate experiments with 3replicates per trial).

Data are plotted as mean ± SD. Data in b and, g-i), analyzed by Log-rank test with 95% c.i, in c-f) and j-l) by unpaired Student’s t-test and in m by ANOVA with 95% c.i.

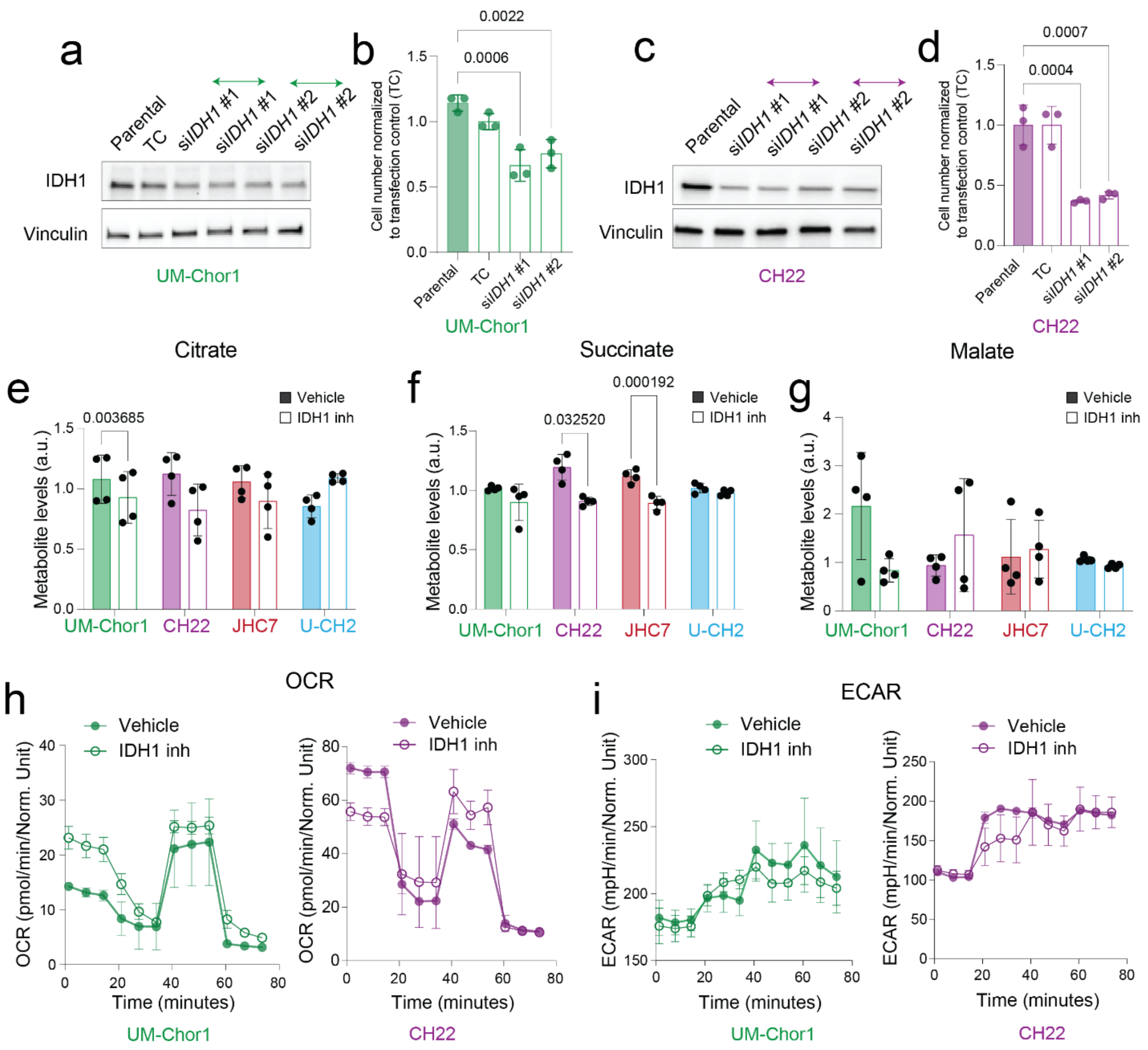

Supplementary Fig. 3 **Targeting IDH1 is toxic to chordoma cells**

a) Representative protein immunoblots for IDH1 (top) and the loading control vinculin (bottom) in the UM-Chor1 chordomas transfected with transfection control or two independent IDH1 siRNA.

b) Cell counts (normalized to transfection control, Y-axis) in UM-Chor1 cells with or without siIDH1 from (n=3 replicates).

c) Representative protein immunoblots for IDH1 (top) and the loading control vinculin (bottom) in the CH22 chordomas transfected with two independent IDH1 siRNA.

d) Cell counts (normalized to parental control, Y-axis) in CH22 cells with or without siIDH1 from (n=3 replicates).

e- g) Relative concentration of citrate (e), succinate (f) and malate (g), (a.u., Y-axis,) measured across four indicated chordoma cell lines (n=4 replicates, each) treated with vehicle (DMSO) or 3μM IDH1 inhibitor for 48 hours prior to metabolite isolation.

h) Seahorse mitochondrial stress test showing oxygen consumption rate (OCR, mPH/min/normalized units, Y-axis) plotted against time (minutes, X-axis) in UM-Chor1 and CH22 treated with vehicle or 3μM IDH1 inhibitor (black line) for 48 hours (n= 6-11 replicates, each).

i) Seahorse of glycolysis stress test showing extracellular acidification rate (ECAR, mPH/min/ normalized units, Y-axis) plotted against time (minutes, X-axis) in UM-Chor1 and CH22 treated with vehicle or 3μM IDH1 inhibitor (black line) for 48 hours (n= 6-11 replicates, each).

Data are plotted as mean ± SD. Data analyzed in b and d using ANOVA, and in e-g by 2-sided, unpaired, 2- tailed, Student's t test, all with 95% confidence intervals.

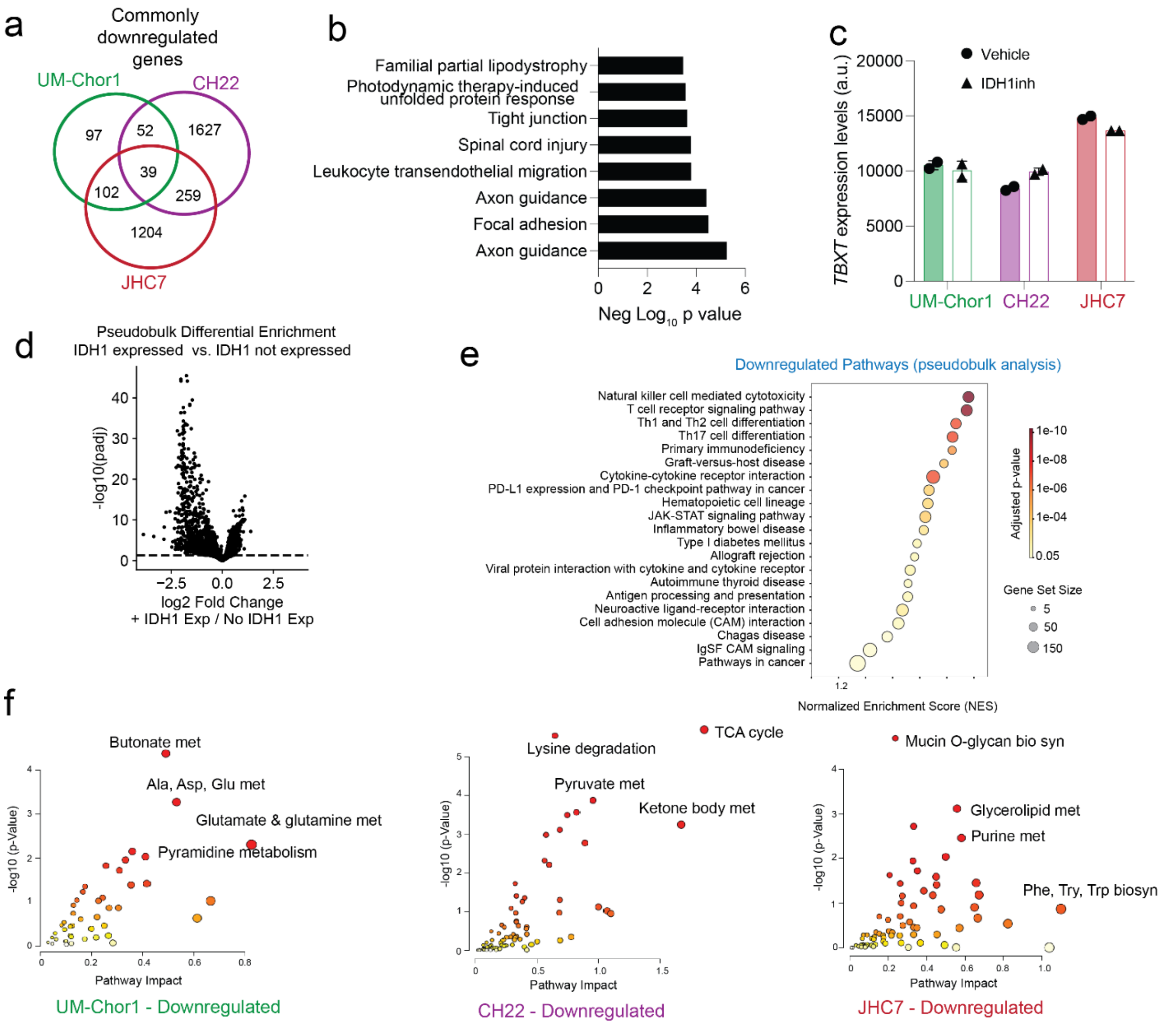

Supplementary Fig. 4 <u>**Glutathione metabolism is a key response pathway to IDH1 inhibition**</u>

a) Three-way Venn diagram showing overlap of genes downregulated by IDH1 inhibition across UM-Chor1, CH22, and JHC7. Shared genes represent consistent transcriptional suppression following IDH1 inhibition.

b) Gene set enrichment analysis (GSEA) of downregulated genes of the overlapping 39-gene gene set from the three tested chordoma cell with pathways illustrated along the Y-axis (negative Log_{10} P-value) for each pathway long the X-axis.

c) Expression levels of the chordoma associated master regulator *TBXT/ BRACHYURY* (Y-axis, a.u.) in IDH1i versus vehicle treated cells across three chordoma cell lines (X-axis).

d) Volcano plot of DESeq2 pseudobulk differential expression comparing IDH1-expressing versus non-expressing tumor cells.

e) KEGG pathways significantly downregulated in IDH1-expressing tumor cells, many involving cytokine and immune-signaling programs.

f) Integrated transcriptomic and metabolic pathway impact analysis of downregulated metabolic pathways for, from left to right, UM-Chor1, CH22, and JHC7 comparing pathway impact (X-axis) versus negative Log_{10} P-value (Y-axis).

Data are plotted as mean ± SD. Data analyzed in c by 2-sided, unpaired, 2- tailed, Student's t test, all with 95% confidence intervals.

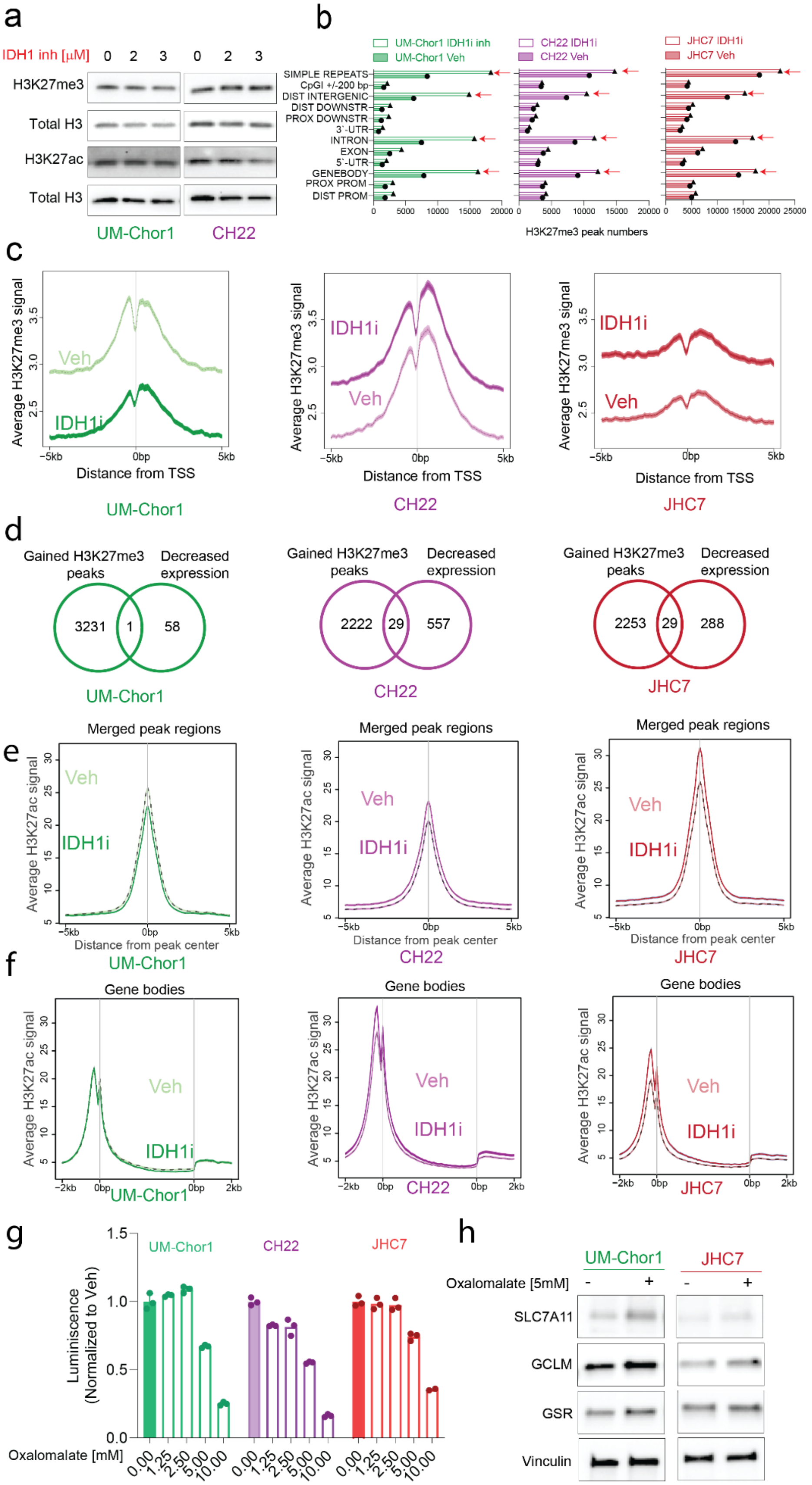

a
IDH1 inh [µM] 0 2 3 0 2 3
H3K27me3
Total H3
H3K27ac
Total H3
UM-Chor1
CH22
b
UM-Chor1 IDH1i inh
UM-Chor1 Veh
CH22 IDH1i
CH22 Veh
JHC7 IDH1i
JHC7 Veh
SIMPLE REPEATS
CpGI +/-200 bp
DIST INTERGENIC
DIST DOWNSTR
PROX DOWNSTR
3`-UTR
INTRON
EXON
5`-UTR
GENEBODY
PROX PROM
DIST PROM
H3K27me3 peak numbers
c
Average H3K27me3 signal
Veh
IDH1i
Distance from TSS
UM-Chor1
CH22
JHC7
d
Gained H3K27me3 peaks
Decreased expression
3231
1
58
2222
29
557
2253
29
288
UM-Chor1
CH22
JHC7
e
Merged peak regions
Average H3K27ac signal
Distance from peak center
f
Gene bodies
Average H3K27ac signal
g
Luminiscence (Normalized to Veh)
Oxalomalate [mM]
0.00 1.25 2.50 5.00 10.00
UM-Chor1
CH22
JHC7
h
UM-Chor1
JHC7
Oxalomalate [5mM] - + - +
SLC7A11
GCLM
GSR
Vinculin

Supplementary Fig. 5 **<u>IDH1 inhibition enriches activating H3K27ac at key glutathione biosynthetic genes</u>**

a) Protein immunoblots for H3K27me3 and H3K27ac with total H3 as loading control in two chordoma cell lines (UM-Chor1 and CH22) treated with vehicle, or 2 µM, or 3µM IDH1 inhibitor for 48 hours.

b) Bar plots showing H3K27me3 peak numbers (Y-axis) at indicated genomic regions in cells treated with IDH1 inhibitor (dark, 3µM for 48hrs) or vehicle (light, DMSO) for three chordoma cell lines (UM-Chor1, CH22, and JHC7 from left-to-right). Red arrows indicate simple repeats, distal intergenic regions, introns, and gene bodies.

c) H3K27me3 composite signal (Y-axis) plots centered at transcription start sites (TSS) of all genes in cells treated with IDH1 inhibitor (dark, 3µM for 48 hours) or vehicle (light, DMSO) for three chordoma cell lines (UM-Chor1, CH22, and JHC7 from left-to-right).

d) Venn diagrams comparing genes associated with increased H3K27me3 signal and genes with decreased transcription in UM-Chor1 (green), CH22 (purple), and JHC7 (blue) chordoma cells.

e) H3K27ac composite signal (Y-axis) plots centered at merged peak regions in cells treated with IDH1 inhibitor (dark, 3µM for 48 hours) or vehicle (light, DMSO) for three chordoma cell lines (UM-Chor1, CH22, and JHC7 from left-to-right).

f) H3K27ac composite signal (y-axis) plots centered at gene bodies in cells treated with IDH1 inhibitor (dark, 3µM for 48 hours) or vehicle (light, DMSO) for three chordoma cell lines (UM-Chor1, CH22, and JHC7 from left-to-right).

g) Cell Titre glo assay, luminiscence counts normalized to a vehicle (DMSO control, Y-axis) across indicated concentrations (X-axis) of Oxalomalate in three different chordoma cell lines, 72 hours after treatment.

h) Protein immunoblotting for SLC7A11, GCLM, GSR with Vinculin as loading control in two chordoma cell lines (UM-Chor1 and JHC7) treated with vehicle, or 5 mM Oxalomalate for 72 hours.

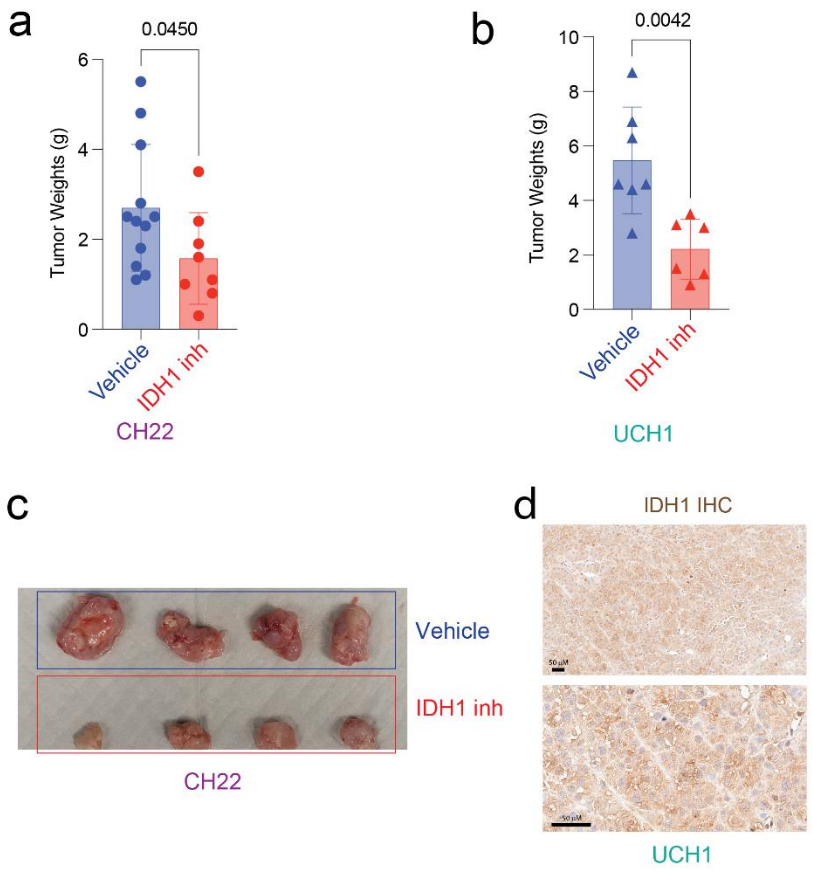

Supplementary Fig. 6 **<u>IDH1 inhibition is therapeutic *in vivo*</u>**

a) Tumor weights for vehicle (DMSO, blue, n=12) or IDH1 inhibitor treated (red, n=11) CH22 flank xenografts at the end of trial (20 days post injection).

b) Tumor weights for vehicle (DMSO, blue, n=7) or IDH1inhibitor treated (red, n=8) UCH1 flank xenografts at the end of trial (26 days post injection).

c) Dissected CH22 tumors from DMSO (top) and IDH1 inhibitor treated (bottom) mice.

d) IHC for IDH1 expression in subcutaneous tumors extracted from mice with UCH1 subcutaneous tumors and treated with vehicle.

Data analyzed in a-b by Mann-Whitney U- test with 95% confidence intervals.
